# From Sea to Sweet: Seaweed's Role in Nutritious and Sustainable Confectionery

**DOI:** 10.1111/1541-4337.70361

**Published:** 2025-12-11

**Authors:** Nima Mohammadi, Nikoo Ostovar

**Affiliations:** ^1^ UCD School of Agriculture and Food Science University College Dublin Dublin Ireland; ^2^ Bioactivity & Applications Laboratory, Department of Biological Sciences, Faculty of Science and Engineering University of Limerick Limerick Ireland

**Keywords:** bioactive compounds, circular economy, innovation, plant‐based, sustainability

## Abstract

Seaweed is increasingly recognized as a multifunctional ingredient in confectionery products, owing to its exceptional nutritional profile, functional properties, and environmental sustainability. This review highlights the integration of seaweed and seaweed‐derived compounds into a wide range of confectioneries, including chocolates, puddings, snack bars, gummies, drinking jellies, and jelly candies. Green, brown, and red seaweed varieties are rich in antioxidants, dietary fiber, essential minerals, and bioactive compounds, such as sulfated polysaccharides, polyphenols, pigments, and phytosterols. These compounds exhibit a range of biological activities, including antioxidant, anti‐obesity, anti‐anemic, and photoprotective effects. Moreover, functional hydrocolloids such as agar, alginate, and carrageenan, derived from seaweed, could substitute animal‐based gelling agents and improve the texture, stability, and shelf life of plant‐based confectionery products. While the inclusion of seaweed enhances the nutritional quality of confectionery products by increasing protein, fiber, and mineral content, challenges persist regarding sensory attributes, formulation optimization, and regulatory approval. Future perspectives should prioritize the application of innovative processing technologies, increased consumer education, clinical trials, and in vivo experiments to substantiate the health benefits of final products, and the exploration of underutilized seaweed species to meet the growing demand for clean‐label, sustainable, circular economy‐aligned, and plant‐based confectionery.

## 1 Introduction

Global food security is increasingly challenged by environmental, socio‐economic, and demographic factors (FAO 2019). While the global population is projected to reach 10.3 billion by the mid‐2080s, putting additional pressure on agricultural resources already constrained by climate‐related limitations (United Nations 2024), food and nutritional insecurity are strongly influenced by socio‐economic and political drivers, including land‐use change, market access, trade policies, and the homogenization of diets. These factors highlight the urgent need to diversify food sources and promote underutilized and nutrient‐rich species, also known as “future smart foods,” to enhance dietary diversity, build resilience, and combat malnutrition (FAO 2018, 2019). Seaweed represents one such underutilized resource with high nutritional value and sustainability potential (Waqas et al. 2024).

Global seaweed production has more than tripled since 2000, reaching 38 million tons by 2022, with approximately 30%–38% used for human consumption (The State of World Fisheries, and Aquaculture, 2024). Seaweed cultivation represents a sustainable alternative (Hofmann et al. 2024), as it does not require pesticides, fertilizers, or freshwater, thereby reducing its environmental footprint (Govaerts and Ottar Olsen 2023). Beyond its eco‐friendly profile, seaweed contributes to food security by providing a nutrient‐dense resource that supports dietary diversification and resilience in the face of declining terrestrial yields (L. Pereira et al. 2024).

Seaweeds are categorized into three main groups: green algae (Chlorophyta), brown algae (Phaeophyta), and red algae (Rhodophyta). Green seaweeds, such as *Ulva*, *Enteromorpha*, *Chaetomorpha*, *Codium*, and *Caulerpa*, contain chlorophyll *a* and *b* as their primary pigments (Cordero 2003). Brown seaweeds, which account for approximately 47% of global seaweed production, contain fucoxanthin, a xanthophyll carotenoid pigment; species such as *Laminaria saccharina* and *Undaria pinnatifida* are examples of brown seaweeds (Purcell‐Meyerink et al. 2021). Red seaweeds, such as *Chondrus crispus*, *Porphyra* sp., and *Gracilaria* sp., owe their distinctive red coloration to carotenoids and phycobiliproteins (Cesário et al. 2018). In addition to their taxonomic diversity, seaweeds are nutritionally rich and increasingly incorporated into functional foods due to their bioactive compounds, dietary fiber, minerals, and vitamins (Thahira Banu and Uma Mageswari 2015a). Recent studies indicate that dietary inclusion of seaweeds may enhance digestive health and reduce the risk of chronic diseases, including diabetes, cancer, and cardiovascular disorders (Meinita et al. 2022; Pradhan et al. 2022).

**FIGURE 1:**
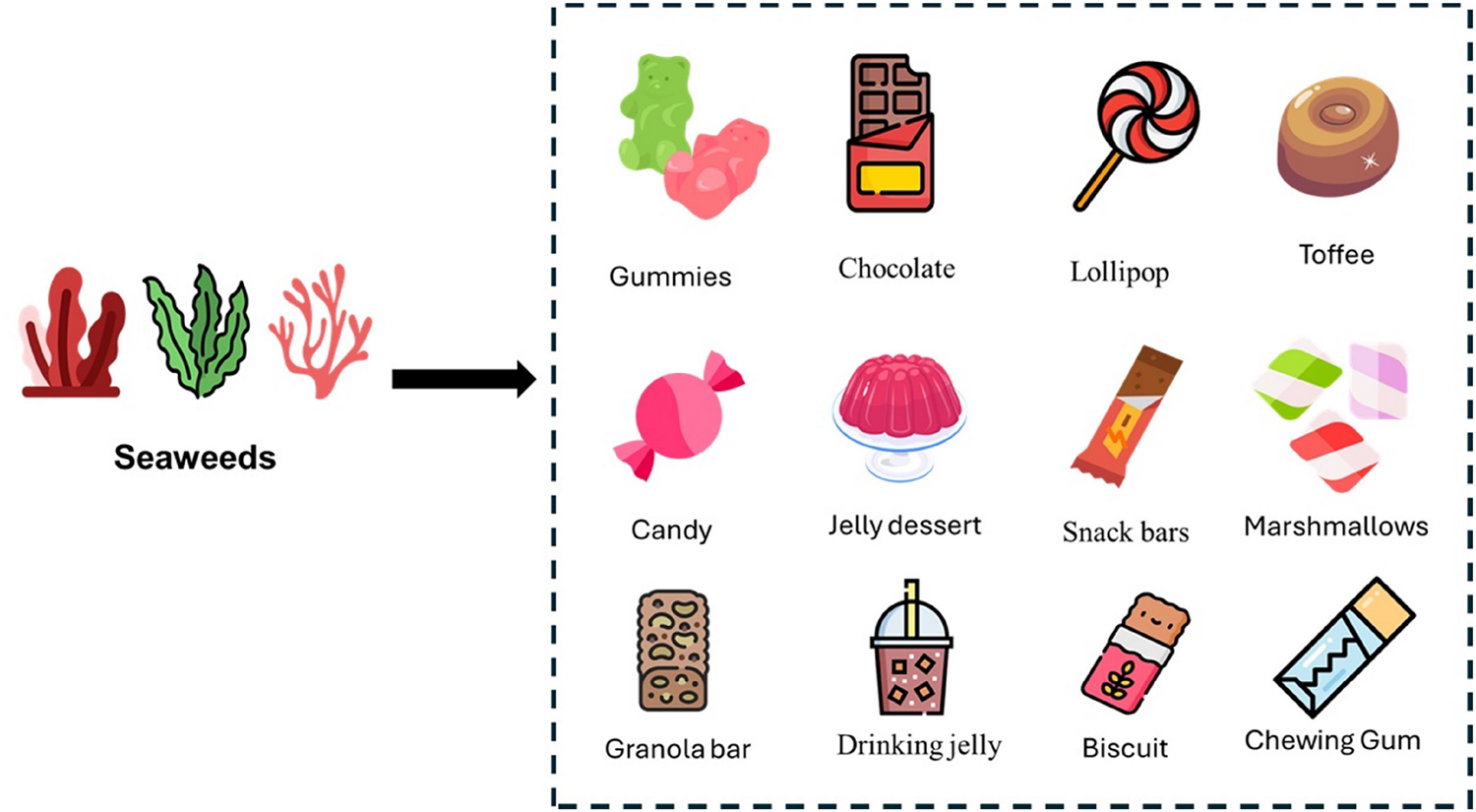
Some examples of confectionery products that can be enriched with seaweed include gummies, chocolates, lollipops, toffee, candies, jelly desserts, snack bars, marshmallows, granola bars, drinking jellies, biscuits, and chewing gums, offering enhanced nutritional and functional benefits.

As consumer demand for clean‐label, functional foods grows, the integration of seaweed and its derivatives into traditionally nutrient‐poor products, such as confectionery, offers a novel strategy to improve nutritional profiles. Traditionally, confectionery products, known for their vibrant colors, appealing shapes, and distinctive flavors (Çoban et al. 2021), have been considered nutritionally poor due to high levels of sugar and fat with minimal essential nutrients (Ivanova et al. 2023). Incorporating seaweed into products such as chocolates, puddings, snack bars, gummies, drinking jellies, and jelly candies offers a novel approach to enhancing their nutritional profile and developing functional foods (Figure 1).

Over the past 10 years, review articles on “seaweeds × functional foods” and “seaweeds health benefits” have primarily focused on the incorporation of seaweeds and seaweed extracts into meat and bakery products (Quitral et al. 2022; Roohinejad et al. 2017). Some studies have also explored the use of seaweed polysaccharides in food packaging (Ali et al. 2024) and examined the health benefits of seaweed‐derived bioactive compounds, including their antioxidant potential (Y. Kumar et al. 2021; Pradhan et al. 2022). However, the use of seaweeds in confectionery products, such as chocolates, puddings, snack bars, gummies, drinking jellies, and jelly candies, and their effects on properties such as color, texture, nutritional value, and sensory attributes remain underexplored. This review addresses that gap by critically examining seaweed's role in reformulating confectionery products. It explores the potential to replace animal‐based ingredients, such as gelatin, with seaweed‐derived alternatives like agar, alginate, carrageenan, seaweed powder, seaweed flour, and their extracts to support a sustainable, plant‐based approach. It also considers reducing refined sugars by incorporating natural or low‐calorie alternatives. These strategies may enhance the nutritional and functional quality of confectionery products by increasing dietary fiber, protein, and minerals, and by enriching them with bioactive phytochemicals from seaweeds. Additionally, seaweed may improve the biological activity of these products through antioxidant, anti‐obesity, anti‐anemia, and photoprotective effects. Seaweed‐enriched, cleaner‐label confectionery products that align with current dietary and sustainability trends could help redefine the category beyond traditional sugary and fatty products.

## 2 Nutritional Composition of Seaweed and Its Impact on Confectionery Products

Seaweeds are low in calories but rich in essential nutrients, including proteins with all essential amino acids, polysaccharides, polyunsaturated fatty acids, and vitamins such as retinol (vitamin A), thiamine (vitamin B1), riboflavin (vitamin B2), and ascorbic acid (vitamin C) (Pradhan et al. 2022). They are also a natural source of essential macronutrients (potassium, magnesium, calcium, sodium, chloride, phosphorus, and sulfur) and trace elements (copper, zinc, selenium, cobalt, nickel, boron, and manganese), with particularly high iodine content, which is crucial for preventing goiter in humans (Pandey et al. 2020). In addition, seaweeds are a source of dietary fiber that resists digestion, is fermented by gut microbiota to produce short‐chain fatty acids, and modulates metabolic pathways, immune responses, and oxidative stress (Huang et al. 2022) (Table 1). Their cell walls also contain hydrocolloids, which impart gelling, thickening, stabilizing, and emulsifying properties, contributing to texture and stability in food systems (Bukhari et al. 2023). In terms of composition, red and green seaweeds generally have higher protein content than brown seaweeds, reaching up to 30% dry weight (d.w.). Green seaweeds tend to be richer in carbohydrates compared to both red and brown varieties. The nutritional composition of seaweeds can differ based on factors like species, size, age, reproductive stage, and the surrounding environment. Environmental factors, like temperature, seawater pH, salinity, depth, nutrient availability, oxygen levels, ultraviolet radiation, light intensity, and herbivory pressure, also influence their nutritional profile.

**TABLE 1:** Nutritional composition of seaweed species across different families (values expressed on a dry weight basis, d.w.b.).

Group	Family	Species (Common name)	Image^a^	Protein (% w/w)	Lipids (% w/w)	Carbohydrates (% w/w)	Dietary fiber (% w/w)	Ref.
Green seaweeds (Chlorophyta)	Ulvaceae	*Ulva lactuca* (sea lettuce)	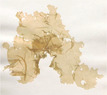	13.60	0.19	58.10	28.40–38.10	(Lahaye 1991; Rasyid 2017)
	Monostromataceae	*Monostroma nitidum* (green laver)	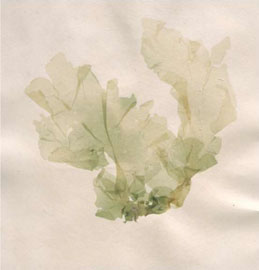	5.10–9.29	0.60–1.82	41.88–46.10	0.94–4.94 (Crude fiber)	(Zhao and Ziyu 2021)
	Caulerpaceae	*Caulerpa* spp. (sea grapes, umibudo)	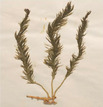	10.41	1.11	38.66	32.99	(Matanjun et al. 2009)
Brown seaweeds (Phaeophyceae)	Fucaceae	*Ascophyllum nodosum* (rockweed)	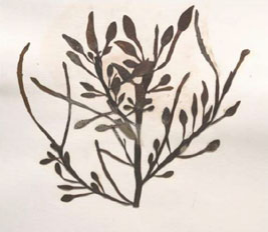	8.70	3.62	27.50	42.60	(Lorenzo et al. 2017; Samarasinghe et al. 2021; Schiener et al. 2017)
		*Fucus vesiculosus* (bladder wrack)	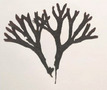	12.99–15.10	3–3.75	56.4	45.00	(Lorenzo et al. 2017; Neto et al. 2018)
		*Fucus serratus* (toothed wrack)	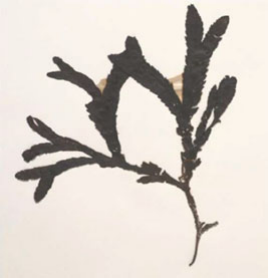	10–17	0.40–3	26–62	16	(Catarino et al. 2018)
		*Fucus spiralis* (spiral wrack)	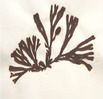	4.14–9.71	4.40–11.54	12.77–17.59	40.44–52.27	(Paiva et al. 2018)
	Alariaceae	*Undaria pinnatifida* (wakame, sea mustard)	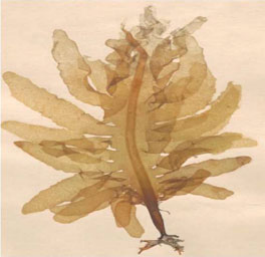	12–14	1	37	14.9–35.30	(Lahaye 1991; Taboada et al. 2013)
	Laminariaceae	*Laminaria digitata* (kelp, oarweed)	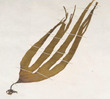	7.75	1.95	69.07	5.50 (Crude fiber)	(Souto‐Prieto et al. 2024)
		*Saccharina japonica* (kombu)	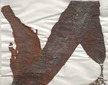	5–11	0.30–2.35	61.48–68.47	29.98	(Deng et al. 2021; Kim 2014; Nie et al. 2022)
		*Saccharina latissima* (sugar kelp)	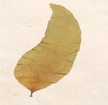	10.20	0.50	68.90	40.90	(Neto et al. 2018)
	Lessoniaceae	*Eisenia bicyclis* (arame)	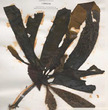	11.10	0.90	54.50	74.60	(Kolb et al. 1999; Lahaye 1991)
	Sargassaceae	*Sargassum fusiforme* (hijiki)	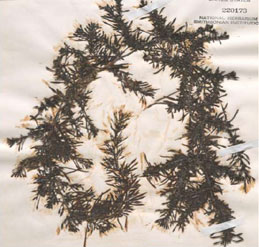	3.90	3.50	14.93	11.30	(Bai et al. 2024; Meinita et al. 2022)
		*Bifurcaria bifurcata* (forked bifurcaria)	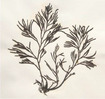	8.92–10.92	5.67‐6.54	40–42	37.42	(Gómez‐Ordóñez et al. 2010; Lorenzo et al. 2017; Mian and Percival 1973)
Red seaweeds (Rhodophyta)	Bangiaceae	*Pyropia tenera* (laver)	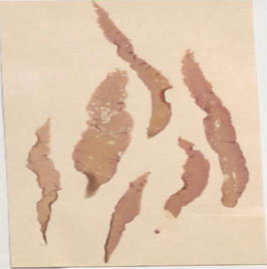	36.83	0.73	—	32.57	(Hwang 2013; Skrzypczyk et al. 2019)
		*Porphyra umbilicalis* (purple laver)	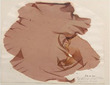	30.36	<0.50	54.51	41.23	(López‐Santamarina et al. 2025)
		*Pyropia yezoensis* (laver)	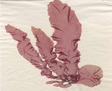	36.20–39.20	2.30–3.10	51.20–57.90	—	(Cho and Rhee 2020)
	Palmariaceae	*Palmaria palmata* (dulse)	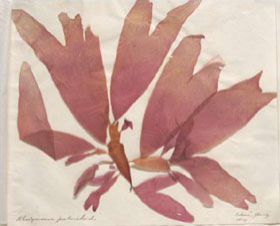	15.61	<0.50	48.70	32.08	(López‐Santamarina et al. 2025)
	Gigartinaceae	*Chondrus crispus* (Irish moss)	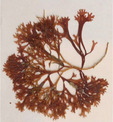	19.47	<0.50	53.72	45.70	(López‐Santamarina et al. 2025)
	Gracilariaceae	*Gracilariopsis lemaneiformis* (ogonori)	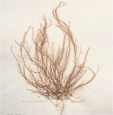	20.87	0.87	61.61	—	(Wen et al. 2006)
	Hapalidiaceae	*Lithothamnion calcareum* (maërl)	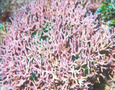	—	—	—	—	—

^a^ Images are from the Seaweeds website: https://www.si.edu/search/.

### 2.1 Carbohydrate Composition

Seaweeds contain a wide variety of carbohydrates such as agar, carrageenan, alginate, ulvan, laminarin, fucoidan, and cellulose/hemicellulose, classified by molecular size as mono‐, di‐, oligo‐, and polysaccharides (L. Xu et al. 2020). Monosaccharide composition in seaweeds varies across different types. In green seaweeds, glucose is the predominant sugar, with rhamnose and arabinose as secondary components. Red seaweeds show the highest diversity, containing sugars such as xylose, galactose, and sorbitol. In brown seaweeds, mannitol is the main monosaccharide, often accompanied by significant amounts of fucose and constitutes about 20%–30% of their dry weight (d.w.), making it valuable for applications in food, chemical, medical, and pharmaceutical industries (Hamid et al. 2019; Hosseini et al. 2024). Mannitol is a six‐carbon, non‐cyclic sugar alcohol and major photosynthetic product in some photosynthetic organisms (Bonin et al. 2015). It plays an important role in antioxidation, carbon storage, osmoregulation, and transport (Hosseini et al. 2024). Mannitol is widely used as a food additive and low‐calorie sweetener, and along with other compounds like triterpenes, it enhances food preservation by reducing sugar crystallization. Some seaweed species, including *Laminaria* and *Saccharina*, are rich in the sugar alcohol mannitol, which can constitute up to 30% d.w. of their composition (Rioux et al. 2017). Additionally, Species like *Sargassum*, *Laminaria*, *Alaria*, *Turbinaria*, *Sacchariza*, *Ulva*, *Kappaphycus*, and *Macrocystis* have been studied for mannitol‐based ethanol bioconversion.

Seaweed‐derived polysaccharides are structurally complex macromolecules composed of multiple monosaccharide units connected by glycosidic bonds. Depending on their monosaccharide composition, they are categorized as either homopolysaccharides or heteropolysaccharides and may exhibit linear or branched configurations. Among seaweed species, polysaccharide content ranges from roughly 4% to 76% d.w., with the highest levels reported in *Ascophyllum* (brown seaweed), as well as *Porphyra* and *Palmaria* (red seaweeds).

Seaweed polysaccharides are usually soluble in water, with certain types containing sulfate groups that contribute to their thickening and gelling capabilities (Akter et al. 2024). Based on their source, they are categorized as Rhodophyta, Fucophyta, or Chlorophyta polysaccharides. Key polysaccharides include agar, alginates, and carrageenan, with additional varieties, such as cellulose, fucoidan, floridean starch, laminarin, ulvan, and xylan. Sulphated polysaccharides like fucoidans are notable for their potential functional applications, while laminarin, a storage polysaccharide, is increasingly recognized for its biofunctional properties.

#### 2.1.1 Seaweed Hydrocolloids

Agar, carrageenan, and alginate are the three primary hydrocolloids commonly used in confectionery products. These hydrocolloids serve as plant‐based substitutes for bovine gelatin, making them suitable for vegan and vegetarian options. Hydrocolloids are long‐chain hydrophilic polymers known for their ability to form viscous dispersions or gels when mixed with water (Abdul Khalil et al. 2018). They are primarily extracted from red (Rhodophyceae) and brown (Phaeophyceae) seaweeds.

Agar is a polysaccharide obtained from red seaweeds like *Gracilaria* and *Gelidium*. It consists of two main components: agarose and agaropectin (Y. Kumar et al. 2021). Agarose is a linear polymer made up of repeating units of 1,4‐linked 3,6‐anhydro‐L‐galactose and 1,3‐linked β‐D‐galactose. Agaropectin, on the other hand, is a sulphated polysaccharide that includes agarose along with additional substances such as D‐glucuronic acid, ester sulphates, and small amounts of pyruvic acid. Agar extracted from specific sources, such as *Gracilaria chilensis*, is particularly suitable for confectionery products with high sugar content including jams, cakes, jellies, chocolates, and fruit candies (Ali et al. 2024). This type of agar is referred to as “sugar‐active” because the presence of sucrose enhances its gel strength (Abdul Khalil et al. 2018). Unlike other hydrocolloids that require potassium or calcium salts for gel formation, agar is often preferred in food applications due to its superior gelling properties and functionality. In many Asian countries, agar is widely used as a plant‐based substitute for gelatin in jellies, where seaweeds are boiled, flavored, and cooled to form the final product. Its applications are primarily determined by functional properties such as gel strength (Abdul Khalil et al. 2018; Pandey et al. 2020). The quality of agar also plays a significant role in its usage; high‐quality agar is preferred for specialized applications, while lower‐quality agar is commonly used in confectionery products like candies, meringues, puddings and dessert gels. Agar's ability to form low gel‐strength media is a unique characteristic that makes it highly adaptable for diverse uses (Ali et al. 2024). Additionally, incorporating agar into cocoa syrups enhances consistency, improves gloss, and stabilizes the system by increasing viscosity and adhesiveness (Sikora et al. 2003). Seaweeds containing agar are utilized in confectionery (10%) and baking (9%) applications (Janke 2024). Beyond these functions, agar also serves as a cryoprotectant and fat substitute, minimizing damage during the thawing and freezing process, and serves as a key component for production of edible films (Ali et al. 2024).

Alginate is a highly effective stabilizer, thickener, and gel‐forming agent (Abdul Khalil et al. 2018; Y. Kumar et al. 2021). Structurally, alginates are linear polysaccharides composed of α‐L‐guluronic acid (G) units and β‐D‐mannuronic acid (M) linked in a (1→4) arrangement, forming an anionic polymeric structure. As a polyelectrolyte, it selectively binds alkaline earth metals like calcium and sodium ions, facilitating gel formation. Due to its ability to chelate metal ions and create highly viscous solutions, alginate is widely used as a stabilizer and thickener in different food products, including jellies, desserts, and ice pops. Alginates are the primary polysaccharides found in the intercellular matrix and cell walls of brown seaweeds (Xie et al. 2024). In brown seaweeds, their content typically ranges from 17% to 47%, with the highest concentrations observed in young blades during July.

Carrageenan is a marine hydrocolloid and serves as the primary structural component of red algae (Xie et al. 2024). Commercially, they are mainly extracted from *Kappaphycus alvarezii* and *Eucheuma denticulatum* (Pujiastuti et al. 2025). The structure of carrageenan consists of linear chains made up of repeating disaccharide units of 3,6‐anhydro‐galactose and D‐galactose, linked through alternating 3‐β‐D‐galactose and 4‐α‐D‐galactose. These chains are further modified by substitutions with methyl, ester sulfate, or pyruvate groups, with sulfate ester content ranging from 15% to 40%, depending on the type of carrageenan (kappa, iota, lambda). It is also widely used in confectionery products, including dessert mousses, gummies, jellies, marshmallows, and bakery fillings, and serves as a stabilizer in ice cream, jams, syrups, instant dessert preparations, and even in honey clarification. For instance, carrageenan derived from red seaweed is commonly used as a thickener in jelly candy (Abdul Khalil et al. 2018; Y. Kumar et al. 2021). Its ability to efficiently bind water helps thicken, stabilize, and enhance the appearance and overall acceptability of confectionery products.

### 2.2 Proteins and Amino Acids

The protein content of red seaweeds varies between 2.7% and 47.0% w/w d.w., with *Laurencia dotyi* Saito having the lowest and *Porphyra tenera* the highest (Rawiwan et al. 2022). Red seaweeds contain all essential amino acids, ranging from 31.1% to 42.1% (g amino acid‐N/100 g protein‐N) in *Grateloupia turuturu* and *Porphyra acanthophora*, respectively. Brown seaweeds generally contain 3% to 15% w/w protein d.w. (Fleurence 2018; Lafarga et al. 2020). However, some species exhibit notably higher protein levels. For instance, *Sargassum vulgare*, *Chnoospora minima*, *Padina gymnospora*, and *Dictyota menstrualis* have protein contents ranging from 10% to 15% w/w d.w., while *Undaria pinnatifida* (wakame) stands out with values between 11% and 24% w/w d.w. Other species, such as *Laminaria hyperborea*, *Alaria esculenta*, and *Saccharina latissima*, typically contain 6.8% to 11% w/w protein d.w. Green seaweeds contain 9% to 26% w/w protein d.w., including glycoproteins, lectins, and phycobiliproteins (Fleurence et al. 2018).

Glutamic and aspartic acids are the predominant amino acids in many seaweed species. Research indicates that these amino acids account for 10% to 15% of the total amino acid content in various red algae species, including *Laurencia flagellifera, Porphyra acanthophora, Aglaothamnion uruguayense*, *Gracilariopsis tenuifrons, Cryptonemia seminervis*, and *Acanthophora spicifera* (Lafarga et al. 2020). Similarly, green seaweed proteins also contain a high proportion of glutamic and aspartic acids (Fleurence 2018). In *Ulva rotundata and U. rigida*, these amino acids can constitute up to 32% and 26% of the total amino acid content, respectively, while in *U. armoricana*, their levels can reach as high as 35%. Brown seaweeds, on the other hand, are particularly rich in amino acids such as lysine, alanine, valine, glycine, leucine, and threonine, while tyrosine, methionine, tryptophan, histidine, and cysteine are present in lower amounts (Lafarga et al. 2020).

### 2.3 Lipids and Fatty Acids

The lipid content of seaweeds typically ranges from 4.6% w/w d.w. in *Enteromorpha clathrate* to 1.33% w/w d.w. in *E. intestinalis* (Pandey et al. 2020). However, some studies have reported lipid levels as high as 12% w/w d.w. in *Utricularia rigida* and as low as 1.09% w/w d.w. in *Kappaphycus alvarezii*. Red and green seaweeds are both valuable sources of polyunsaturated fatty acids (PUFAs), including EPA and DHA, with red seaweed offering a favorable omega‐6/omega‐3 ratio of 0.8 (Gamero‐Vega et al. 2020; J. Xu et al. 2023).

### 2.4 Minerals

Seaweed typically has a high ash content, reflecting its substantial mineral composition, with minerals constituting up to 36% d.w. (Rajapakse and Kim 2011). The macronutrients found in seaweed include sodium, potassium, calcium, sulfur, magnesium, phosphorus, and chlorine, while its micronutrients consist of iron, iodine, boron, zinc, selenium, copper, nickel, molybdenum, manganese, fluoride, and cobalt. A study on edible seaweeds reported that brown varieties contain the highest mineral levels among green, red, and brown groups, particularly potassium (118 mg/g d.w.), calcium (37.9 mg/g d.w.), sodium (98.4 mg/g d.w.), magnesium, selenium (31.7 mg/g d.w.), zinc (18 mg/g d.w.), chromium (55 mg/g d.w.), and iodine (21,000 mg/g d.w.) (Rajapakse and Kim 2011). Red seaweeds contained the highest levels of manganese (0.360 mg/g d.w.) and copper (15.8 mg/g d.w.), while green seaweeds had the highest concentrations of phosphorus (30 mg/g d.w.) and iron (9.43 mg/g d.w.). This highlights brown seaweeds as the most mineral‐rich group, followed by red and green seaweeds with specific mineral advantages.

### 2.5 Vitamins

Seaweeds are a rich source of both fat‐soluble vitamins A, cholecalciferol (D), tocopherols (E), and phylloquinone (K), and water‐soluble vitamins such as C and the B‐complex group (Xie et al. 2024). The vitamin content in seaweeds can vary based on species, seasonal changes and environmental conditions (Hagan and Anyangwe 2023). These vitamins play key roles in maintaining healthy skin, hair, nails, supporting immune system, as well as connective tissue (Polat et al. 2023). While seaweeds are not particularly high in vitamins pantothenic acid (B5), pyridoxine (B6), biotin (B7), folate (B9), and E, they contain significant amounts of vitamins B1, B2, and cobalamin (B12). Vitamin B2 is essential for energy metabolism, especially in the breakdown of proteins. Importantly, seaweeds are among the rare non‐animal sources of vitamin B12, which is crucial for red blood cell production, DNA synthesis, and neurological health. Also, levels of vitamin B12 have been identified in various seaweed species, including *Pylaiella littoralis, Fucus vesiculosus* (a brown seaweed), and *Ulva lactuca* (a green seaweed), highlighting seaweed's potential as a valuable nutrient source, particularly for plant‐based diets (Susanti et al. 2022).

### 2.6 Effect of Adding Seaweed or Its Derivatives on Nutritional Composition of Confectionery Products

In efforts to promote consumer wellness and align with clean label trends, researchers and the food industry are actively reducing sugar and saturated fat levels in confectionery products and exploring the use of seaweed and their derivatives to enhance the nutritional quality, functional properties, and sensory attributes of these products. For example, seaweed powder derived from the red seaweeds *Kappaphycus alvarezii* and *Gracilaria edulis* was incorporated into dark chocolate at varying concentrations, while a control sample was prepared without seaweed (Debbarma et al. 2024). The composition of seaweed‐enriched chocolate was analyzed in triplicate, assessing protein, lipids, ash, mineral content (sodium and potassium), carbohydrates, moisture, water activity (*a*_w_), and energy value. The results showed that incorporating red seaweed increased the protein content of dark chocolate, with chocolates containing 7.5% *K. alvarezii* and 7.5% *G. edulis* exhibiting higher protein content than the control. Additionally, the inclusion of *G. edulis* and *K. alvarezii* significantly reduced fat content compared to the chocolate control, with decreases ranging from approximately 7.67% to 22.69% for *G. edulis* and 2.82% to 23.57% for *K. alvarezii*. Nevertheless, it is still unclear how seaweed ingredients affect fat in the product, whether by displacing cocoa butter or by interacting with lipids. A detailed study of texture and flow could help explain if seaweed polysaccharides interfere with fat crystallization, which would influence both texture and fat content in the final product. The study also showed that fortifying dark chocolate with seaweed increased ash content in all samples, with chocolates containing *G. edulis* ranging from 1.95% to 2.49%, and those with *K. alvarezii* ranging from 2.62% to 4.39%. Seaweed‐enriched chocolate exhibited a noticeable increase in Na and K content in all samples, except for Na in the 1% *K. alvarezii* chocolate, where the increase was not significant. This is likely because the low seaweed concentration (1%) was insufficient to cause a meaningful change in nutrient levels. As a result, including this small amount of seaweed may not be effective. While the mineral increase corresponded with higher ash content, the study did not assess their bioavailability. Future research should use in vivo trials to determine whether the elevated sodium and potassium levels provide physiological benefits or pose potential risks. Chocolate fortified with 2.5% and 5% *G. edulis* showed a substantial increase in carbohydrate content, whereas all samples containing *K. alvarezii* exhibited a significant decrease compared to the control. Yet, the study did not specify the monosaccharide or oligosaccharide composition. Without sugar profiling, it is unclear whether the increased carbohydrates are nutritionally beneficial, such as contributing to dietary fiber. The water activity and moisture content of both the control sample and seaweed‐enriched chocolate ranged from 0.42 to 0.44 and 0.60% to 1.80%, respectively. Due to their low *a*_w_ and moisture content, these chocolate products were considered to have a low risk for microbial growth. Furthermore, the energy value of seaweed‐fortified chocolates was notably lower than that of the control sample. However, the study did not report lipid oxidation values or sensory shelf‐stability over time, which are critical given the fatty matrix of chocolate.

In another study, researchers also incorporated aqueous extracts of unidentified brown seaweed into jelly candy at concentrations of 5%, 10%, and 15%. (Faridah 2019). After measuring the carbohydrate, lipid, protein, ash, and moisture content of the optimal formulation based on sensory results (15% extract), it was found that protein and ash contents were significantly higher, with the increased ash level attributed to the high mineral content of brown seaweed. Surprisingly, no significant differences were observed in the carbohydrate, lipid, and moisture content compared to the control. The lack of change in carbohydrate and lipid content suggests that aqueous extraction selectively isolates water‐soluble components (such as proteins, minerals, and some polysaccharides) while excluding hydrophobic components. Moreover, the type of seaweed used can influence the final product formulation. For example, if the aim is to reduce fat content or develop fat‐free products, aqueous extracts of seaweed may be appropriate. On the other hand, if the goal is to enrich the product with carbohydrates, adding dried seaweed powder to products like dark chocolate, as demonstrated in the study by Debbarma et al., could be effective.

In a study, *Eucheuma cottonii* flour, which contains 69.3% dietary fiber, was incorporated into milk chocolate bars at varying levels (0%, 5%, 10%, and 15%) to analyze its impact on water and fiber content (Stefani et al. 2019). The water content was measured by evaporating moisture through heating, while the fiber content was determined by treating the sample with an alkaline solution to isolate coarse fiber from other components. Fiber content analysis revealed a significant increase in dietary fiber levels with the addition of *E. cottonii* flour. The fiber content in milk chocolate bars rose from 1.32% in the control sample to 8.65% in the chocolate containing 5% seaweed flour. Similarly, another study explored the addition of different formulations of *E. cottonii* seaweed with tofu dregs to increase the fiber and protein content in chocolate pudding (Sukotjo et al. 2020). Formulations containing 15% tofu dregs and 15% seaweed extract, as well as 25% seaweed extract and 5% tofu dregs, showed the highest protein content (3.92%) and the highest dietary fiber content (5.38%), respectively. Both studies using *E. cottonii* seaweed extract increased the fiber content of the final products. However, *E. cottonii* flour, compared to the extract, even in small amounts, significantly increased the fiber content more than the seaweed extract. While these increases in nutritional markers are promising, the reliance on incorporating high amounts (up to 15%–30%) may compromise product quality and cost‐effectiveness. Focusing on optimizing functional compounds, such as sulfated polysaccharides, rather than bulk additions, could yield similarly improved nutritional value with less impact on the final product formulation.

One study found that incorporating dried powder of *Caulerpa racemosa*, commonly recognized as sea grape, into biscuits enhanced their nutritional value (A. Kumar et al. 2018). The analysis measured protein, moisture, ash, and carbohydrate content. Protein profiling of the samples revealed distinct bands associated with eight peptide chains, with estimated molecular weights ranging from 20 to 116 kDa. Increasing the amount of *C. racemosa* in the biscuits resulted in a rise in protein content from 7.69% to 9.01%, while ash content also rose from 1.28% to 2.23%. In contrast, carbohydrate levels decreased from 71.7% to 68.75%. A slight reduction in lipid content was also observed, although it was not statistically significant. Biscuits supplemented with 10% *C. racemosa* contained higher protein and mineral (ash) content. Additionally, the fiber content increased significantly from 0.30% to 1.83%, *as C. racemosa* is a notable source of dietary fiber (13%). However, a biscuit with only 1.83% fiber is still considered low compared to the minimum required to make a “source of fiber” nutrition claim, which is 3 g fiber/100 g (Food Safety Authority of Ireland 2021). Aligning product formulations with these regulatory benchmarks would help clarify commercial feasibility and support legally compliant health or nutrition claims. Similarly, researchers added *Caulerpa* sp. seaweed extract to candy formulations using extract‐to‐water ratios of 0:4, 1:3, 2:2, 3:1, and 4:0. The chemical analysis of the seaweed candy exhibited changes in ash, moisture, and protein composition, and dietary fiber content depending on the extract‐to‐water ratio. Moisture ranged from 11.75% to 17.08%, while protein content varied between 34.32% and 39.38%. The unusually high protein levels (34%–39%) in the candy are noteworthy and likely reflect the use of a highly concentrated extract or a substantial contribution from gelatin. The highest ash content was observed in the 4:0 ratio. Reducing sugar content was also influenced by the extract level, increasing from 2.39% at the 2:2 ratio to 4.37% at the 1:3 ratio. Dietary fiber content increased with higher extract concentrations, ranging from 0% to 8.31%, highlighting the candy's potential as a fiber‐rich product. Overall, the chemical composition depended on the extract‐to‐water ratio used during preparation, suggesting that formulation significantly influences the nutritional quality of the final product (Fransiska et al. 2020).

Since nutritional profiles vary widely by species, a thorough compositional fingerprint including proximate, micronutrient, and polysaccharide profiling should precede formulation to ensure alignment between expected results and product goals. Seaweed powders typically boost fiber and reduce fat but may introduce pigments and off‐flavors, making them more suitable for fiber fortification. Aqueous extracts, in contrast, could be better suited for enhancing protein and mineral content without affecting sweetness. These distinct functional contributions highlight the importance of standardized extract characterization. Without clear profiling of bioactive fractions, reproducibility and scalability remain significant challenges.

### 2.7 Seaweed‐Derived Hydrocolloids in Confectionery Products

Seaweed‐derived hydrocolloids, such as agar, carrageenan, and alginate, are widely used in confectionery for their gelling, thickening, and stabilizing properties, offering plant‐based alternatives to gelatin. These hydrocolloids gel through three primary mechanisms: cold‐set, heat‐set, and ionotropic gelation (Saha and Bhattacharya 2010). For instance, agar and κ‐carrageenan undergo cold‐set gelation, forming gels as the temperature decreases (Avallone et al. 2023), whereas alginate gels via ionotropic gelation in the presence of calcium ions (Saha and Bhattacharya 2010). In this context, a study explored the potential of agar extracted from *Gracilaria corticata* and *Gracilaria edulis* for plant‐based food jellies (De Alwis and Wijesekara 2022). The jellies were made by dissolving dried agar powder, sourced from *G. corticata* and *G. edulis*, in water, with a concentration of 1.5% w/v. The results indicated *G. corticata* showed a higher pH (6.70) than *G. edulis* (6.38), which may affect viscosity. Viscosity decreased with temperature for both species, but *G. edulis* had significantly higher values at 70°C and 80°C, suggesting stronger molecular interactions. Gelling temperature was slightly higher in *G. corticata* (38.1°C) than *G. edulis* (36.4°C), while *G. edulis* exhibited a higher melting temperature (60.6°C vs. 54.3°C), indicating greater thermal stability. Syneresis (water loss from gel) was significantly lower in *G. corticata* (2.75%) than in *G. edulis* (29.7%), indicating a more stable gel structure with improved water retention during jelly storage. Factors like agar concentration and gel strength influence syneresis, as previously reported by Menaka and Wijesekara (2025). In their study, *Gracilariopsis longissima* (formerly *G. verrucosa*) was processed to extract agar, a gelling agent commonly used as a gelatin substitute in the food industry (Menaka and Wijesekara 2025). Agar was extracted at different temperatures (90°C, 70°C, and 50°C), with the highest yield of 40.25 g/100 g dry seaweed obtained at 90°C. The functional properties of the agar, including gelling and melting temperatures, viscosity, pH, and syneresis, were evaluated. Agar extracted at 90°C showed the highest gelling and melting temperatures, stronger gel formation, and the best gel strength, resulting in less syneresis. Lower extraction temperatures led to reduced viscosity. Thus, the gelling properties of agar were influenced by the extraction method. Additionally, different amounts of agar (4, 8, and 12 g) were added to plant‐based jellies, with increasing agar concentration resulting in firmer textures. Jellies containing 12 g of agar were the firmest, followed by those with 8 and 4 g, showing a progressive increase in hardness.

Regarding the use of carrageenan in confectionery products, research demonstrated its significant impact on product quality. For example, adding different concentrations of red seaweed carrageenan (3.5% and 5%) and cinnamon powder (0.5%, 1%, and 1.5%) to jelly candy significantly influenced its gel strength, texture, and water‐binding capacity (Setiawan et al. 2024). The formulation with 1.5% cinnamon and 5% carrageenan achieved the highest water content (22.84%) and elasticity (2.76%), while the lowest values were observed in the 0.5% cinnamon, 3.5% carrageenan formulation (11.94% water content, 1.90% elasticity). This is due to carrageenan's ability to bind large amounts of water and create a stable structure. At higher concentrations, however, excessive water retention can soften the gel matrix, reducing elasticity as the excess water diffuses into the gel network and weakens its structural integrity (Atmaka et al. 2013). These results align with those of Stefani et al. (2019), who demonstrated that adding *E. cottonii* flour to milk chocolate bars increased the water content from 1.66% in the control sample to 2.1% in chocolate containing 5% seaweed. They highlighted that the increase in water retention was attributed to carrageenan due to its strong water‐binding properties. Thus, while carrageenan enhances gel strength and texture, its water‐binding capacity must be carefully balanced (Setiawan et al. 2024).

Alginate can form ionic gels only in the presence of multivalent cations, a characteristic commonly used for encapsulating active ingredients in the food industry (Bi et al. 2022). Among these cations, calcium is the preferred choice due to its low toxicity and suitability for food applications, leading to the widespread use of calcium alginate gels. However, this requirement for specific ions limits its flexibility, making alginate less commonly used in confectionery products than more versatile gelling agents like agar and carrageenan.

Despite these limitations, alginate has long been used in traditional Chinese cuisine to prepare jelly dishes, owing to its gel‐forming properties (Qin 2018). Traditionally, such jellies are made by cooking seaweeds such as *Gelidium amansii* and *Sargassum pallidum*, which release gel‐forming substances that solidify into a hydrogel upon cooling. In more modern applications, sodium alginate is combined with slow‐releasing calcium compounds to produce heat‐stable jellies, which can be served as cold dishes or stir‐fried with other ingredients. The formation and stability of alginate gels depend on several factors, including pH, the mannuronic/guluronic acid (M/G) ratio, molecular weight, temperature, and calcium concentration. Controlling the pH is crucial for preventing alginate degradation, while temperature regulation is important, as alginate gels are thermo‐irreversible (Lee and Mooney 2012). The M/G ratio also plays a significant role: higher guluronic acid content results in stronger gels. Additionally, alginates with lower molecular weight tend to form more stable gels with reduced syneresis (Pournaki et al. 2024). The calcium‐to‐alginate ratio further affects gel texture and stability. An excess of calcium can lead to syneresis, whereas insufficient calcium may result in weak gels (Lee and Mooney 2012). For instance, alginate‐based edible dessert jellies typically exhibit a firmer texture and do not melt like gelatin jellies, which soften and dissolve at body temperature (Qin et al. 2018). This difference is largely attributed to the specific combinations of calcium salts and sodium alginate, which set at varying rates depending on the dissolution speed of the calcium salt.

Seaweed‐derived hydrocolloids such as agar, carrageenan, and alginate offer plant‐based gelling alternatives with distinct functional properties suited to various confectionery products. Agar demonstrates strong gel formation and thermal stability, with its performance influenced by extraction temperature and concentration. Carrageenan enhances gel strength, texture, and moisture retention in confectionery products, but its high water‐binding capacity must be carefully balanced to avoid softening the gel structure. In contrast, alginate forms heat‐stable gels through ionotropic gelation, offering controlled texture and reduced syneresis. Nonetheless, its dependence on multivalent cations limits its flexibility in confectionery applications. Overall, these hydrocolloids present versatile options for formulating plant‐based jellies, with optimal functionality depending on precise control of formulation and processing conditions.

## 3 Bioactive Phytochemicals in Seaweed and Their Functional Roles in Confectionery

Seaweeds are rich in bioactive compounds, such as sulfated polysaccharides, polyphenols, pigments, and phytosterols, offering various health benefits, including antioxidant, anti‐inflammatory, antimicrobial, antitumor, anticoagulant, antiviral, and anti‐diabetic effects. The addition of seaweed extracts to confectionery products presents an innovative approach to creating functional foods with improved antioxidant, anti‐obesity, anti‐anemia, and photoprotective properties (Table 2). Several studies have emphasized the nutritional and bioactive benefits of seaweed‐enriched confections, underlining their contribution to health and their potential to address specific nutritional and medical issues (Farida et al. 2022; Liu et al. 2024; Thahira Banu and Uma Mageswari 2015a).

**TABLE 2:** Comparison of biological activities in seaweed‐enriched confectionery studies.

Biological activity	Seaweed type	Confectionery type	Extract/compound used	Observed effects	Ref.
Antioxidant capacity	*Fucus vesiculosus*	Granola bars	Water, ethanol, and acetone extracts	Reduced lipid oxidation; DPPH scavenging; metal chelation; reducing power	(Hermund, Karadaǧ, et al. 2016; Karadağ et al. 2017)
	Brown seaweed (Nirwana coast)	Jelly candy	Brown seaweed extract	Increased phenolic content; improved DPPH scavenging	(Faridah 2019)
	*Undaria pinnatifida*	Seaweed gummies	Fucoxanthin‐enriched extract	DPPH scavenging activity; storage stability	(Liu et al. 2024b)
	Atlantic wakame (AW), Sea lettuce (SL)	Seaweed gummies	AW and SL extracts	AW gummies had the highest TPC; commercial gummies had the highest FRAP	(Xavier et al. 2024)
Anti‐obesity activity	*Eucheuma cottonii*	Snack bars	Seaweed, beetroot	High fiber content; potential for obesity management	(Farida et al. 2022)
Anti‐anemia activity	*Ulva reticulata*	Chocolate	Seaweed extract	Improved iron status in anemic girls	(Thahira Banu and Uma Mageswari 2015b)
Photoprotective activity	*Undaria pinnatifida*	Seaweed gummies	Fucoxanthin‐enriched extract	UVB protection; enhanced cell viability	(Liu et al. 2024b)

### 3.1 Sulfated Polysaccharides

Sulfated polysaccharides are complex, negatively charged carbohydrates located within the cell walls of seaweeds, which are mainly composed of cellulose and hemicellulose (Lomartire and Gonçalves 2022). With a high carbohydrate profile and minimal fat content, these compounds possess various bioactivities such as antimicrobial, antioxidant, anticoagulant, anti‐inflammatory, antitumor, and antiviral properties. Unlike terrestrial plants, seaweed‐derived dietary fibers uniquely contain sulfated polysaccharides with varying sulfate group compositions. (Huang et al. 2022). Seaweed‐derived dietary fibers are classified into soluble and insoluble types. Soluble dietary fibers include laminarin, alginate, and fucoidan in brown seaweeds; agar, carrageenan, and agarose in red seaweeds; and ulvan and xylans in green seaweeds. Insoluble dietary fibers, on the other hand, consist of lignin, cellulose, hemicellulose, and starch, most of which exhibit limited bioactive properties. The total dietary fiber content in seaweed varies between 36% and 60% d.w., with approximately 55%–70% of this fiber being soluble (Rajapakse and Kim 2011). Among seaweeds widely utilized in the food industry, *U. pinnatifida*, *Porphyra* and *Chondrus* have the highest soluble fiber content, while *Fucus* and *Laminaria* contain the highest levels of insoluble fiber. The recommended average daily intake of dietary fiber is about 28 g in the United States, 25 g for women and 38 g for men in Canada, 25 g in the European Union, and more than 18 g in the United Kingdom (Miller 2020).

Fucoidan is a sulfated polysaccharide with diverse biological activities, attributed to its variable sulfate group (Y. Kumar et al. 2021). Structurally, it is a polymer of fucan sulfate, primarily composed of 1,2‐linked L‐fucose‐4‐sulfate units. In some cases, it also includes 1,3‐ or 1,4‐linked fucan sulfate chains with side branches containing galactose, uronic acid, and xylose residues. It exhibits anticancer, anticoagulant, antiviral, immunomodulatory, anticomplement, antiproliferative, and antithrombotic effects. Fucoidan is typically extracted from brown seaweeds such as *Saccharina longicruris*, *Ecklonia cava*, *Fucus vesiculosus*, *Undaria pinnatifida*, and *Ascophyllum nodosum*. Fucoidans predominantly consist of fucose and sulfate ester groups, along with other sugar components like mannose, glucose, galactose, acetyl groups, xylose, and uronic acids (Xie et al. 2024). As anionic polysaccharides, fucoidans serve as key structural components of brown seaweed cell walls and are absent in terrestrial plants. Their content in brown seaweeds usually ranges from 10% to 20%, with the highest recorded concentration of 46.6% in *Laminaria digitata*. Despite being discovered over a century ago, the complete chemical structure of fucoidan remains unresolved. Unlike alginate, fucoidan lacks gelling and thickening properties (A. Kumar et al. 2023; Rioux et al. 2017); nevertheless, it holds potential for novel food applications and is utilized as an active ingredient in biomedical, food, and pharmaceutical industries.

Laminarans are a food storage polysaccharide with a simpler chemical structure compared to fucoidans (Y. Kumar et al. 2021). They consist of linear glucans and mannitol with β‐1,3 linkages. It exists in two forms: soluble laminaran from *Laminaria hyperborea* and insoluble laminaran from *Laminaria digitata*. Depending on mannitol content, laminarans are classified into M‐chains, with mannitol at the reducing end, and G‐chains, with glucose at the terminal region. Laminaran exhibits various biological activities, including antiapoptotic, immunomodulatory, and antitumor effects.

Ulvans are unique sulfated polysaccharides found exclusively in green seaweeds of the *Ulva* genus and are well known for their potent antioxidant properties (Xie et al. 2024). They are primarily composed of uronic acids (glucuronic and iduronic) and neutral monosaccharides such as glucose, rhamnose, and xylose. The main repeating disaccharide units, known as aldobiouronic acids, include β‐D‐glucuronosyl‐(1→4)‐α‐L‐rhamnose 3‐sulfate. In some cases, sulfated xylose may substitute for the uronic acids. The structure of ulvans is heterogeneous, with characteristic repeating units such as A3s [→4)‐β‐D‐glucuronic acid‐(1→4)‐α‐L‐rhamnose‐3‐sulfate‐(1→] and B3s [→4)‐β‐L‐iduronic acid‐(1→4)‐α‐L‐rhamnose‐3‐sulfate‐(1→]. Sulfate groups typically occupy the C‐3, or both the C‐1 and C‐3 positions, on the rhamnose residue. Ulvans constitute up to 40% d.w. of *Ulva* species and represent the dominant soluble dietary fibers, alongside minor components such as xylo‐glycans and glucuronans.

### 3.2 Polyphenols

Seaweeds are valuable sources of diverse polyphenolic compounds known for their wide‐ranging biological properties. Prominent polyphenols in seaweeds include bromophenols, phlorotannins, phenolic acids, phenolic terpenoids, and flavonoids (Zhao et al. 2023). Phlorotannins, found exclusively in brown seaweeds, are polymers of phloroglucinol and are structurally categorized into six types: fucols, eckols, carmalols, fucophloretols, fuhalols, and phloretols (Santos et al. 2019). Their concentration is influenced by species, geographical factors, and seasonal variation, with *Ascophyllum nodosum* and *Fucus vesiculosus* showing notably high levels (5.80%) (Connan et al. 2004). On the other hand, bromophenols, particularly tribromophenol, are more common in red and green seaweeds, with red seaweeds containing 8–2590 ng/g and green seaweeds 0.9–2393 ng/g (Shannon et al. 2021). In brown seaweeds, bromophenol levels are lower, ranging from 2 to 454 ng/g. These compounds serve as ecological defense agents. Other phenolic compounds include gallic acid, identified in *Himanthalia elongata*, and catechin, a predominant flavonoid (Luna‐Guevara et al. 2018). These compounds are recognized for their potent antioxidant, anti‐inflammatory, antimicrobial, anticancer, and antidiabetic activities (Zhao et al. 2023).

Several studies explored adding phlorotannins from brown seaweeds to confectionery products. Phlorotannins were extracted from the brown algae *Sargassum ilicifolium* and encapsulated in a chitosan–tripolyphosphate coating. This encapsulation helped address the challenges posed by phlorotannins, such as their low bioavailability, bitter taste, astringency, dark brown color, unfavorable odor, and loss of activity during processing and storage. After encapsulation, the phlorotannins were incorporated into the gel. The bioactivity retention of encapsulated and non‐encapsulated phlorotannins was investigated through in vitro gastrointestinal digestion and colon fermentation. Encapsulated phlorotannins in gel showed better bioavailability and controlled release, maintaining higher levels during later digestion phases. Non‐encapsulated phlorotannins in gel had higher antioxidant and anti‐diabetic effects in early stages, but encapsulation enhanced activity in colonic fermentation. Overall, encapsulating phlorotannins in gel improved their delivery, enhanced their bioactive potential, and masked their unfavorable taste (Kaushalya and Gunathilake 2022).

### 3.3 Pigments

Carotenoids are natural pigments, ranging from yellow to orange‐red, that contribute to the coloration of marine organisms, particularly brown seaweeds (Miyashita et al. 2020). These tetrapenoid compounds are essential for photosynthesis and are classified into two main categories: xanthophylls, such as astaxanthin, zeaxanthin, lutein, and fucoxanthin, which contain oxygen atoms, and carotenes, such as lycopene, α‐carotene, and β‐carotene, which are hydrocarbons (Gomes et al. 2022). Among these, fucoxanthin, a key xanthophyll in brown seaweeds, makes up about 10% of natural carotenoids (Pangestuti and Kim 2011). Additionally, *β*‐carotene is most abundant in brown and green seaweeds, with lower concentrations in red seaweeds (Nwachukwu et al. 2016). Lutein is primarily found in green seaweeds, with smaller amounts in red seaweeds, while zeaxanthin is present in green and red seaweeds (Gomes et al. 2022). The color, bioavailability, and chemical reactivity of carotenoids are influenced by their isomeric forms, molecular polarity, and functional groups (Cooperstone et al. 2015). Carotenoids also exhibit a wide range of bioactivities, including antioxidant, antitumor, anti‐diabetic, antiviral, anti‐obesity, and anti‐inflammatory effects, while also protecting against chronic diseases and UV‐induced damage (Maeda 2015; Pérez‐Gálvez et al. 2020).

Seaweeds contain four types of chlorophyll, which are greenish pigments, with chlorophylls *a*, *b*, and *c* being the most prevalent. Chlorophyll *a*, the most abundant and widely distributed, has a blue–green hue and plays a central role in electron transport, energy transmission, and light absorption (Manzoor et al. 2024). Chlorophyll *b*, which appears green–yellow, is found exclusively in green seaweeds, while chlorophyll *c*, present in brown seaweeds, includes types *c*1 and *c*2, with *c*1 being the most common (Jeffrey 1972; Manivasagan et al. 2018). Red seaweeds typically contain chlorophyll *a* (Kato et al. 2020). Chlorophylls are essential pigments in photosynthetic organisms due to their tetrapyrrole ring structure with a central magnesium ion and a hydrophobic tail, making them highly reactive to light and heat (Aryee et al. 2018). Chlorophylls exhibit bioactivity through their antioxidant, antimutagenic, and anticarcinogenic effects. Their unique structure allows them to scavenge free radicals, prevent DNA damage, and regulate disease‐related cellular processes, while their hydrophobic side chains facilitate membrane interactions, enhancing uptake and signaling (Martins et al. 2023). This antioxidant capacity is attributed to chlorophyll *a*, which improves the activity of vitamin E (Pérez‐Gálvez et al. 2020).

Phycobiliproteins are water‐soluble, non‐toxic fluorescent proteins found in rhodophyta, where they make up to 60% of the soluble proteins. These proteins include phycocyanin, allophycocyanin, phycoerythrocyanin, and phycoerythrin, all of which play key roles in photosynthetic light harvesting (Cotas et al. 2020). The red color of Rhodophyta is due to phycoerythrin, with R‐phycoerythrin being the most abundant in red seaweeds (Rossano et al. 2003). Phycocyanin, a blue light‐harvesting pigment, is another important protein, while allophycocyanins are located in the core of phycobilisomes. Marine red seaweeds, such as *Porphyridium cruentum*, produce phycobiliproteins, which can constitute up to 8% of their total composition (Ghosh et al. 2022). Due to their antioxidant capacities, free‐radical scavenging abilities, and fluorescence properties, phycobiliproteins are widely used in food, cosmetics, biomedical, and pharmaceutical industries (T. Pereira et al. 2020).

### 3.4 Phytosterols

Phytosterols are fatty compounds found in seaweeds, resembling cholesterol in structure but differing in their C_24_ side chains. They are essential components of cell membranes, with their composition varying depending on the type of seaweed (Sohn et al. 2021). Brown seaweeds are particularly rich in brassicasterol and fucosterol, making them a valuable source of phytosterols. In contrast, red seaweeds primarily contain cholesterol, with smaller amounts of phytosterols like desmosterol and sitosterol. Green seaweeds show a species‐dependent range of sterols, including *β*‐sitosterol, ergosterol, and poriferasterol (Ling and Jones 1995; Sánchez‐Machado et al. 2004). Phytosterols are recognized as safe for human consumption by regulatory bodies like the FDA and EU, with no reported toxicity (Dutta 2003). Additionally, phytosterols such as fucosterol, saringosterol, and ostreasterol offer a broad range of health benefits, including cholesterol reduction, anticancer, anti‐inflammatory, and neuroprotective properties, making them promising candidates for functional food development (Chen et al. 2023).

### 3.5 Biological Activity of Seaweed‐Enriched Confectionery Product

#### 3.5.1 Antioxidant Capacity

Researchers assessed the antioxidant capacities of water, 80% ethanol (v/v), and 70% acetone (v/v) extracts of *Fucus vesiculosus* in granola bars enriched with 5% fish oil (Hermund, Karadaǧ, et al. 2016; Karadağ et al. 2017). Ethanol and acetone extracts (0.5 or 1 g/100 g emulsion) reduced lipid hydroperoxide formation over a 10‐week storage period at 20°C, while the water extract showed no effect (Karadağ et al. 2017). Ethanol and acetone extracts exhibited the 1,1‐diphenyl‐2‐picryl hydrazil (DPPH) radical scavenging activity (100.7% and 101.5%, respectively), metal chelation (52.3% and 28.9%, respectively), and reducing power (1.6 for both). The antioxidant capacities of the ethanol and acetone extracts were linked to their high total phenolic content (TPC) (ethanol extract: 20.4 g gallic acid equivalents [GAE]/100 g dry extract; acetone extract: 23.2 g GAE/100 g dry extract) and the strong interfacial affinity of phenolic compounds, which may enhance tocopherol regeneration (Karadağ et al. 2017). In addition, their high phlorotannin content, which is the primary phenolic compound in brown algae (Hermund et al. 2016), contributes to their antioxidant activity. It should be noted that all these measurements rely exclusively on in vitro chemical assays. Therefore, adding seaweed to granola bars or any type of confectionery product does not guarantee that their consumption will provide antioxidant activity. In other words, without in vivo data, such as animal or human studies tracking oxidative biomarkers after consumption, it is premature to claim functional health advantages. Notably, in vivo results from another study on 32 adult rats over a 30‐day period confirmed the antioxidant activity of *Fucus vesiculosus* (Eshtiwi 2024). The researchers found that daily supplementation with 1 cm^3^ of 150 mg/cm^3^ extract from *Fucus vesiculosus* significantly reduced glutathione peroxidase (GPx) activity and peroxynitrite (ONOO^−^) levels in serum. The decrease in ONOO^−^ suggests reduced oxidative stress, whereas the lowered GPx activity may reflect a disruption in the antioxidant defense system. More recent work has highlighted the role of seaweed‐derived polysaccharides, particularly carrageenan, in enhancing antioxidant stability. In jelly candy, carrageenan encapsulates phenolic compounds within a hydrophilic gel network, preserving their activity during heat processing and storage (Setiawan et al. 2024). The hydroxyl groups in carrageenan facilitate double helix formation, further stabilizing bioactive compounds. Notably, higher concentrations of carrageenan lead to increased antioxidant capacities, underscoring its dual role as both a texturizer and a preserving agent. The dual role of carrageenan as both a texturizer and a protective agent is interesting. Despite this, the result is still based on in vitro studies. How phenolics and carrageenan‐bound complexes behave during gastrointestinal digestion has yet to be explored. Similarly, fucoidan from the brown seaweed *Fucus vesiculosus* exhibits a multi‐faceted antioxidant mechanism, involving hydrogen donation, electron transfer, and structural factors such as sulfate content, molecular weight, and polyphenol presence (Pozharitskaya et al. 2020). in vivo results demonstrated that fucoidan treatment at a dose of 300 mg/kg body weight, administered intragastrically daily for 8 weeks, exerted significant antioxidant activity in a mouse model of ethanol‐induced liver injury (Xue et al. 2021). Fucoidan markedly reduced oxidative stress, which was typically elevated during ethanol metabolism and contributed to associated oxidative damage. This protective effect was mediated through activation of a key cellular energy regulation and antioxidant signaling pathway, which enhanced the liver's endogenous antioxidant defenses and improved mitochondrial function. Consequently, this modulation decreased lipid peroxidation and restored redox balance, effectively mitigating ethanol‐induced oxidative injury in the liver.

In another study, jelly candy with 15% brown seaweed extract from the coast of Nirwana in West Sumatra had a higher TPC (360 mg GAE/g fresh weight [FW]) than the control (301.5 mg GAE/g FW). in vitro, antioxidant capacity followed a similar trend, with IC_50_ values based on the DPPH assay at 3.2 and 2.7 mg/L, respectively (Faridah 2019). These results further confirmed that incorporating brown seaweed extract into jelly candy enhanced its in vitro antioxidant activity. It remains to be determined whether this benefit translates to in vivo conditions, such as enhanced antioxidant enzyme activity or reduced DNA and lipid oxidation markers in human subjects (Trigo et al. 2023), remains unaddressed. Similarly, researchers aimed to develop fucoxanthin‐enriched seaweed gummies using *Undaria pinnatifida* pulp to evaluate their antioxidant properties (Liu et al. 2024). Seaweed gummies containing 0%, 0.5%, 0.75%, and 1% fucoxanthin were analyzed under accelerated storage conditions for up to 16 days. The gummy sample without fucoxanthin exhibited a lower DPPH scavenging rate of less than 15%. In contrast, the DPPH scavenging rates for the 0.5%, 0.75%, and 1% fucoxanthin‐enriched seaweed gummies were 55%, 60%, and 70.66%, respectively. By the end of the accelerated storage period (day 16), the DPPH scavenging rates had declined to 4.54%, 18.24%, 29.70%, and 43.76% for the 0%, 0.5%, 0.75%, and 1% fucoxanthin‐enriched seaweed gummies, respectively. Similarly, a study revealed that fucoxanthin extracted from *Undaria pinnatifida* using ethanol exhibits dose‐dependent DPPH scavenging activity (Lourenço‐Lopes et al. 2023). These in vitro data showed that fucoxanthin concentrations directly influence in vitro radical‐scavenging activity (Iwasaki et al. 2012). In contrast, in vivo studies involving the feeding of female mice with 0.1% fucoxanthin over 3 weeks showed that fucoxanthin exhibited antioxidant activity through indirect metabolic modulation rather than direct antioxidant action. Therefore, assessing the antioxidant activity of seaweed in final products should involve both in vitro chemical assays and in vivo models to ensure a comprehensive evaluation. Researchers also formulated gummies with Atlantic wakame (AW) and sea lettuce (SL) extracts (23 g/100 g of the final product) (Xavier et al. 2024). TPC analysis showed that AW gummies had the highest content (1.83 mg GAE/30 g), followed by SL gummies (1.62 mg GAE/30 g) and the control gummies (1.61 mg GAE/30 g). Additionally, ferric‐ion reducing antioxidant capacity (FRAP) values were higher for AW gummies (0.94 mg AAE/30 g) and the control gummies (0.77 mg AAE/30 g) compared to SL gummies (0.68 mg AAE/30 g) (Xavier et al. 2024). This cross‐species comparison is valuable, but all antioxidant measurements remain in vitro. Importantly, feeding Wistar rats 50 mg/kg body weight of SL every other day for 10 weeks demonstrated strong in vivo antioxidant activity by decreasing lipid peroxidation and boosting antioxidant enzyme levels in rats with chemically induced breast cancer (Abd‐Ellatef et al. 2017). Similarly, researchers developed a functional jelly using *Eisenia bicyclis* extract enriched with dieckol, a type of phlorotannin (Lim et al. 2024). The extraction process achieved a high yield of 16.5 mg/g biomass under optimal conditions (55.3% ethanol, 70.9°C, 87.3 min). The jellies were formulated to provide 25%–100% of the recommended daily intake (9.954 mg) of dieckol. As the concentration of the extract increased, antioxidant capacity enhanced, as evidenced by the rising ABTS (2,2'‐azinobis(3‐ethylbenzothiazoline‐6‐sulfonic acid)) values, which increased from 0.02 to 0.40 mg AAE/mL. This increase was attributed to dieckol, a key phlorotannin known for its strong antioxidant properties. Among the formulations, the jelly containing 25% of the recommended dieckol intake achieved the best balance between antioxidant capacity and consumer acceptability. Moreover, in vivo studies demonstrated that dieckol downregulated the expression of inducible nitric oxide synthase (iNOS, which produces nitric oxide linked to oxidative stress and tissue damage) and cyclooxygenase‐2 (COX‐2, an enzyme responsible for the synthesis of pro‐inflammatory prostaglandins involved in pain and inflammation) under high‐glucose conditions in adult zebrafish, thereby confirming its antioxidant and anti‐inflammatory effects (E. A. Kim et al. 2015).

Fortifying jellies and gummies with seaweed extracts such as phlorotannins, fucoidan, and fucoxanthin offers potential for developing functional products with enhanced antioxidant capacity. However, most studies rely on in vitro assays like DPPH, FRAP, and ABTS, which do not fully reflect physiological conditions and overlook digestion, absorption, metabolism, and bioavailability in humans. Therefore, future research should prioritize well‐designed in vivo studies, particularly controlled human trials, to validate health claims and antioxidant activity under realistic conditions.

#### 3.5.2 Anti‐Obesity Activity

Researchers evaluated the addition of *Eucheuma cottonii* seaweed and beets (*Beta vulgaris* L.) in snack bars designed for obese elementary school children (Farida et al. 2022). The seaweed contains a relatively high amount of soluble fiber (18.25 g/100 g). The formula was created to meet the nutritional needs of a low‐energy diet and was formulated in isocaloric proportions, offering 1500 kcal of energy, 20% fat, 15% protein, and 65% carbohydrates. The final formulation of the snack bars contained fiber levels ranging from 1.93 to 2.12 g per 100 g. The optimal treatment level consisted of a 10:44:25 ratio of wheat flour, seaweed, and beet in 100 g, providing 209.86 kcal of energy, 2.12 g of fiber, 3.08 g of protein, 42.67 g of carbohydrates, and 2.99 g of fat. These results are in agreement with an in vivo study that demonstrated dietary supplementation with 5% *E. cottonii* for 16 weeks in male rats fed a high‐cholesterol, high‐fat diet significantly mitigated obesity‐related parameters (Matanjun et al. 2010). Rats that received the high‐cholesterol, high‐fat diet supplemented with *E. cottonii* exhibited reduced body weight gain and adiposity compared to the untreated group receiving only the high‐cholesterol, high‐fat diet, despite comparable food intake. These results suggested that *E. cottonii* exerted anti‐obesity effects, potentially through modulation of lipid metabolism and energy balance, supporting its potential application in confectionery products for obesity management.

#### 3.5.3 Anti‐Anemia Activity

A study was conducted to evaluate the nutritional status and create a product based on *Ulva reticulata* green seaweed to address anemia in adolescent girls (Thahira Banu and Uma Mageswari 2015a). A total of 500 girls, aged 15–18, participated, with 100 exhibiting moderate anemia (hemoglobin levels between 7–9 g/dL) and receiving supplementation for 120 days. A chocolate enriched with seaweed, providing 56 mg of iron per 100 g and 11.80 mg of bioavailable iron, was developed and evaluated for its in vitro bioavailability. The results indicated a significant improvement in hemoglobin (8.53 ± 0.27 to 9.60 ± 0.59 g/dL), total iron‐binding capacity (TIBC) (from 397.84 ± 18.1 to 423.38 ± 12.36 mg/dL), mean corpuscular hemoglobin (MCH) (22.49 to 27.59 µg), mean corpuscular volume (MCV) (74.47 ± 2.61 µm^3^ to 84.39 ± 3.37 µm^3^), and serum iron (23.20 to 27.88 µg/dL). Similarly, the anti‐anemia study involved four types of seaweed, *Ulva* sp., *Gracilariopsis* sp., and *Porphyra* sp., and *Sargassum* sp., tested on individuals aged 18 to 45 years with mild to moderate anemia. Participants consumed daily doses of 5 to 20 g over a period of 4 to 8 weeks. The results indicated notable increases in hemoglobin levels and improved iron absorption in all groups. Among the seaweeds, *Sargassum* and *Gracilariopsis* were effective even at lower doses (5 to 10 g), whereas *Porphyra* needed higher amounts (up to 20 g) to show comparable effects. No side effects were observed, and cooking did not reduce the iron bioavailability of the seaweeds (García‐Casal et al. 2007). Similarly, iron content increased nearly fivefold, from 26.4 to 126 mg/100 g, in Pakoda, a traditional Indian snack, as the concentration of *Enteromorpha compressa* added to the snack increased (Mamatha et al. 2007). The bioavailability of iron in Pakoda containing 7.5% *Enteromorpha* was similar to that in *Enteromorpha* alone at pH 7.5 (intestinal condition), with both showing around 55%–56% bioavailability. Under gastric conditions (pH 1.35), however, the bioavailability of iron in the Pakoda was slightly higher (27.1%) than in *Enteromorpha* alone. These results suggest that both seaweed‐enriched chocolate and snacks could serve as effective food supplements for tackling iron deficiency.

#### 3.5.4 Photoprotective Activity

The study on fucoxanthin‐enriched seaweed gummies made with *Undaria pinnatifida* pulp demonstrated their potential in protecting retinal Müller cells from ultraviolet B (UVB)‐induced damage (Liu et al. 2024). Different concentrations of fucoxanthin‐enriched seaweed gummies (0%, 0.5%, 0.75%, and 1%) were tested, with the 0.5% fucoxanthin gummies showing the highest cell viability (71.65% on day 1), while higher concentrations were less effective. Although the protective effect slightly decreased over seven days, the gummies still contributed to reducing UVB‐induced oxidative stress. These eye‐care gummies may improve cell viability, indicating their potential as photoprotective agents. Fucoxanthin protects against UVB‐induced damage by scavenging reactive oxygen species (ROS) and quenching singlet oxygen, thereby reducing oxidative stress and photoaging (Pangestuti et al. 2018). It also stimulates the filaggrin gene, enhancing skin barrier function. When consumed orally, as in the study by Liu et al., fucoxanthin and its metabolites are absorbed and distributed to tissues, providing additional protection to the skin and eyes. Moreover, an in vivo study demonstrated that application of fucoxanthin offers effective photoprotection (Rodríguez‐Luna et al. 2018). Applied at 200 µg per site daily, starting two days before and continuing until 48 h after a single UVB exposure (360 mJ/cm^2^), fucoxanthin protected the skin of hairless mice. It improved skin hydration and elasticity, decreased melanin levels (which can cause uneven pigmentation) and epidermal thickening (a sign of skin damage), and reduced inflammation markers, such as edema (swelling) and cyclooxygenase‐2 (COX‐2) expression. Additionally, fucoxanthin enhanced antioxidant defenses by increasing nuclear factor erythroid 2‐related factor 2 (Nrf2) and heme oxygenase‐1 (HO‐1) protein levels, which help protect skin cells from oxidative damage, supporting its strong anti‐inflammatory and antioxidant effects. Additionally, fucoidan, a sulfated polysaccharide derived from *Undaria pinnatifida*, has demonstrated significant photoprotective effects against UVB‐induced skin damage. This is primarily achieved by inhibiting matrix metalloproteinase‐1 (MMP‐1), an enzyme responsible for collagen degradation, through the suppression of the nuclear factor kappa B (NF‐κB) signaling pathway, which plays a central role in inflammation and skin ageing (Pangestuti et al. 2018). Similarly, in vivo application of low molecular‐weight fucoidan at doses of 0.2, 1.0, and 2.0 mg/cm^2^ for 15 weeks effectively protected mice from UVB‐induced skin damage (Y. I. Kim et al. 2018). The highest dose (2.0 mg/cm^2^) proved to be the most effective. Fucoidan significantly reduced wrinkles and swelling, lowered inflammation and oxidative stress, protected collagen by inhibiting matrix metalloproteinases, and prevented skin cell death.

The incorporation of seaweed extracts into confectionery products offers a promising strategy for diversifying this traditionally sugar‐rich category with functional food attributes. This can be achieved by formulating reduced‐sugar or sugar‐free products and enriching them with seaweed or seaweed‐derived compounds. Recent efforts to reformulate confectionery products into more nutrient‐dense options have gained scientific interest due to the bioactive potential of seaweed components, such as phlorotannins, fucoxanthin, sulfated polysaccharides, and essential minerals, which have been associated with antioxidant, anti‐obesity, anti‐anemia, and photoprotective effects. Several studies have shown that seaweed‐enriched confectionery products, including granola bars, gummies, jelly candies, and snack bars, can enhance nutritional profiles and exhibit improved bioactivity in vitro. Despite these promising results, most evidence is derived from in vitro assays and limited animal studies, highlighting the need for further in vivo research and well‐designed human clinical trials to confirm physiological relevance, bioavailability, and sustained efficacy. Balancing functional efficacy with sensory acceptability remains a key challenge in product development. While seaweed‐fortified confectionery products have the potential to serve as clean‐label alternatives that align with evolving consumer demands for healthier snacks, any health claims must be substantiated through rigorous clinical evidence across diverse populations.

## 4 Effect of Adding Seaweed or Seaweed‐Derived Ingredients on Color, Texture, and Sensory Properties of Confectionery Products

Sensory evaluation is crucial in assessing consumer acceptance and demand for new functional food products, particularly those that may be unfamiliar to certain populations, such as seaweed in many European countries. Therefore, it is important to develop a product that aligns with consumer expectations by refining its sensory attributes (Salgado et al. 2023; Sasue et al. 2023). For instance, reducing or eliminating a component from a standard product formulation can negatively impact its appeal. Likewise, adding a component based on functionality studies may significantly alter the product's texture or sensory characteristics (Konar et al. 2016). In the case of confectionery products, the color, flavor, and texture are critical for consumer acceptance and success (Table 3). Furthermore, in the production of clean‐label food products, edible pigments extracted from seaweeds can be used as natural colorants, offering a safer alternative to synthetic dyes (Mamat et al. 2024). The primary photosynthetic pigments in microalgae include carotenoids (such as fucoxanthin in brown seaweed), chlorophylls, phycobilins, phycoerythrin, and phycocyanin. For example, *Ulva lactuca* (green algae) and *Gracilaria verrucosa* (red algae) contain carotenoids, chlorophyll, and phycobiliproteins like phycoerythrin and phycocyanin (Jacob‐Lopes et al. 2020). These pigments are water‐soluble and heat‐sensitive, making them suitable replacements for artificial dyes (Jayasinghe et al. 2016). A key advantage is their safety, as they have been consumed for generations without adverse effects. They also provide essential minerals, vitamins, antioxidants, and anti‐inflammatory benefits, making them ideal for incorporation into aqueous food systems. However, their drawbacks include lower tinctorial strength and stability, which are generally inferior to artificial dyes.

**TABLE 3:** Seaweed impact on confectionery color, texture, and sensory attributes.

Seaweed type/ingredient	Confectionery product	Color effects	Texture effects	Sensory scores	Key notes	Study reference
Atlantic wakame with fucoxanthin (0%–1%)	Gummies	L* stable; b* stable; a* ↑, less green.	Hardness ↑, chewiness ↑ over time; springiness ↓ less with fucoxanthin	0.5% highest for flavor, shape, color/appearance ↓ at higher concentrations.	Fucoxanthin stabilized texture	(Liu et al. 2024a)
Atlantic wakame, sea lettuce extracts (23 g/100 g); artificial dyes	Gummies	Control: L*, a*, b*, C* ↑; wakame redder, sea lettuce greener; L* ↓.	No difference; similar to control	All rated “liked slightly” (≈6/9 hedonic scale); a*, b* altered by pigments.	Natural pigments were less intense	(de Lima Xavier et al. 2024)
Kombu (4%), nori (3%), sea lettuce (3%)	Chocolates	Kombu highest color rating, then nori, sea lettuce.	Kombu: firm, elastic (alginate gel)	≈7/9 for appearance, color, texture; kombu highest color score.	Alginate enhanced kombu texture	(Salgado et al. 2024)
*Ulva lactuca*, *Sargassum wightii* pigments	Jelly dessert	Matched artificial colors.	Matched artificial‐colored samples	Similar to artificial colors, natural pigments are preferred for safety	Pigments safer, less stable	(Jayasinghe et al. 2016b)
*Gracilaria edulis*, *Kappaphycus alvarezii* (1%–7.5%)	Dark chocolate	L* stable; a*, b* positive.	*G. edulis*: hardness ↑; *K. alvarezii*: inconsistent hardness	Color changes accepted; no appearance impact	Processing reduced fishy smell	(Debbarma et al. 2024)
*Gracilaria* sp. with mango juice (8.93%–16.07%)	Jelly candy	12.5%–16.07% preferred	Control best; others similar	16.07% highest odor/flavor; control lowest flavor; color best at 12.5%–16.07%.	Mango juice boosted sensory appeal	(Luthfiah et al. 2024)
*Gracilaria fisheri* (0.2%–1.0%)	Strawberry drinking jelly	L*, a* ↑; b* ↓; 0.8% optimal	Gel strength ↑; 0.8% optimal; 1.0% over‐gelled ↓	0.8% highest for color, texture, flavor balance	Agarans enhanced gelling	(Charoenphun et al. 2025)
*Eucheuma cottonii* with butterfly pea	Jelly	Drying temps ↓ chlorophyll, L*; purplish tint	50°C best gelatinous texture; higher temps ↓ density	60°C highest taste; texture varied by temp (3.80/5 at 50°C).	Carrageenan shaped texture	(Sukmawati et al. 2024)
*Eucheuma cottonii* with red ginger (0%–60%)	Jelly candy	Appearance stable.	Chewy, dense, sandy (carrageenan, sugar).	50% ginger highest taste, aroma; masked seaweed smell.	High carrageenan formed strong gels.	(Amalia et al. 2021)
*Eucheuma cottonii* flour (0%–15%)	Milk chocolate bar	—	5% highest rated	5% highest overall; 10%–15% ↓ preference; taste > texture > appearance	Low seaweed optimized appeal	(Stefani et al. 2019)
*Eucheuma cottonii*, tofu dregs (5:25–25:5)	Chocolate pudding	15:15 ratio preferred	15:15: dense, soft (carrageenan)	15:15 highest texture, color, aroma; taste similar to 5:25, 10:20	Carrageenan ensured gel consistency	(Sukotjo et al. 2020)
Brown seaweed (0%–15%)	Jelly candy	15% most intense	—	15% highest color, flavor, acceptability (3–4/4 scale)	Fucoxanthin, alginate boosted appeal	(Faridah 2019)
*Caulerpa* sp. (seaweed:sugar 1:0.5–1:1.5)	Jelly candy	1:1 greenest (chlorophyll).	1:1.5: elastic, solid	1:1.5 highest taste, texture, scent; sweet‐salty balance	Higher sugar reduced saltiness	(Tapotubun et al. 2018)
*Caulerpa* sp. extract (0:4–4:0)	Candy	—	2:2 balanced	2:2 highest texture, flavor, aroma; mint/guava masked odor.	Moderate extract optimized appeal.	(Fransiska et al. 2020)

Researchers measured the color attributes, including brightness (L*), redness to greenness (a*), and yellowness to blueness (b*), of *Atlantic wakame (Undaria pinnarifida)* pulp seaweed gummies enriched with different concentrations of fucoxanthin (0%, 0.5%, 0.75%, and 1%) (Liu et al. 2024). The samples were sliced to a thickness of 5 mm and analyzed using a colorimeter. The addition of fucoxanthin to seaweed gummies had no significant impact on L*, with values between 60.65 and 61.28, or on b*, which ranged from 12.95 to 12.98. However, a* slightly increase in a concentration‐dependent manner, moving from −1.11 to −0.52. Consequently, the reddish‐brown tint of fucoxanthin likely contributed to a modification in the color attribute of the seaweed gummies, particularly reducing their greenness. They observed that low concentrations of fucoxanthin resulted in a color like that of the control group (which did not contain fucoxanthin). The results of the sensory evaluation of these gummies by 20 panelists showed that as the concentration of fucoxanthin increased, sensory scores for color and appearance declined, likely due to the gummies' darker color. Seaweed gummies containing 0.5% fucoxanthin received the highest ratings for flavor, shape, and overall sensory attributes. Furthermore, the texture profile analysis of fucoxanthin‐enriched seaweed gummies showed that storage time influenced their texture. Hardness and chewiness increased over time, likely due to water loss, while high temperature and humidity led to a reduction in springiness. However, gummies containing fucoxanthin exhibited less noticeable changes. This could be attributed to fucoxanthin's ability to form hydrogen bonds with κ‐carrageenan, creating a compact network that retains moisture and helps maintain springiness (Qiu et al. 2024). Therefore, fucoxanthin enhanced structural stability, maintaining the gummies' texture during storage.

A similar study incorporated Atlantic wakame and sea lettuce seaweed extracts (23 g/100 g) into gummies, using organic honey and xylitol as sweeteners to replace sugar in the functional product (de Lima Xavier et al. 2024). Control gummies were made with sucrose and an artificial green color solution (Green S E142 and Tartrazine E102, 0.5 g/100 g), and the final product's color was measured using a handheld spectrophotometer. Their results showed that the control gummy had the highest values for color intensity (C*), L*, a*, and b*. Atlantic wakame and sea lettuce gummies exhibited moderate to lower lightness, with Atlantic wakame appearing redder and sea lettuce greener. Both also differed in b* and C*. These results were consistent with the study by Liu et al., who showed that natural pigments from seaweed impacted gummy color, particularly in modifying a* and b*, while having a minimal effect on L*. Regarding the texture of the gummies, there were no notable differences in texture preference, with all samples receiving an average rating of “liked slightly.” This study found that the seaweed gummies had a texture similar to that of the control gummies. However, Liu et al. reported that increased seaweed extract concentrations led to a firmer and more elastic texture in jelly candy. This discrepancy could be linked to the different gelling agents used, as well as the use of honey and xylitol as sweeteners. In the study by de Lima Xavier et al., gelatin (10 g/100 g) may have masked any texture modifications caused by the seaweed extract, whereas Liu et al. used 2% κ‐carrageenan, which may have interacted differently with the extract in the gummy formulation. Other researchers also conducted a sensory evaluation of three different chocolates: milk chocolate with 4% kombu (MK), ruby chocolate with 3% nori (RN), and white chocolate with 3% sea lettuce (WS) (Salgado et al. 2024). The study involved 366 participants aged between 18 and 34 years old, who used a 9‐point hedonic scale (1 = “dislike extremely” to 9 = “like extremely”). The results showed that all chocolate samples received favorable sensory acceptance for appearance, color, and texture, with many participants giving average liking scores around 7. However, no instrumental texture data (such as hardness or chewiness) accompanied the sensory scores, making it difficult to confirm whether polysaccharides directly influenced texture. Among the samples, MK received the highest average score for color, followed by RN and WS in descending order. This might be attributed to the fact that alginate is the primary polysaccharide found in the cell walls of kombu. In brown algae such as kombu, the cell wall structure is reinforced by a network of alginate gel and cellulose, along with components like fucoidan and glycoproteins. In the presence of calcium, alginate forms cross‐links, producing calcium alginate, a gel that is insoluble, elastic, heat‐resistant, and capable of withstanding freezing. This contributes to the firm, elastic texture of kombu (Kato et al. 2016). Furthermore, researchers found that the addition of sea lettuce to the products can significantly impact their color, appearance, aroma, and taste, mainly due to its high chlorophyll content and the presence of amino acids, which contribute to the distinctive aroma through the formation of volatile compounds (Sinurat et al. 2024).

In another study, researchers extracted and purified chlorophyll and carotenoid pigments from *Ulva lactuca* (sea lettuce, a green seaweed) and *Sargassum wightii* (a brown seaweed) using acetone (80%), methanol (90%), and ethanol (90%) for use in jelly desserts (Jayasinghe et al. 2016). The highest values for each pigment and solvent type were as follows: Chlorophyll *a* was highest in *U. lactuca* with 60.7 mg/g FW using 90% methanol; Chlorophyll *b* was highest in *U. lactuca* with 54.8 mg/g FW using 90% methanol; and carotenoids were highest in *S. wightii* with 54.7 mg/g FW using 90% methanol. The extraction yields were impressive, but the use of high concentrations of toxic solvents like methanol raises concerns about residual solvent in food applications. After incorporating these pigments into the jelly dessert, sensory evaluation and texture analysis showed that samples with artificial colors had similar scores to those containing naturally extracted seaweed pigments. While the comparable sensory scores were favorable, the study did not address color stability over time, including pigment degradation and fading. Natural pigments are often prone to deterioration under light and heat, which can impact shelf life. Nevertheless, since synthetic colorants have been linked to health issues such as irritability, hyperactivity, allergies, and even cancer with long‐term exposure, natural pigments are preferred as safer additives or indicators (Manzoor et al. 2024).

In another study, researchers analyzed the color and texture of dark chocolate containing 1%, 2.5%, 5%, and 7.5% (w/w) *Gracilaria edulis* and *Kappaphycus alvarezii* dried red seaweed powder using Hunter's colorimeter (Debbarma et al. 2024). The incorporation of red seaweed in chocolate influenced its color but did not significantly alter L*, with all samples resembling the control (commercial chocolate). Both seaweed‐enriched chocolates and the control exhibited positive a* and b* values. The sensory panel accepted the color changes, suggesting that seaweed could be added to dark chocolate without affecting its appearance. It is obvious that higher concentrations of seaweed contain larger amounts of pigments and a strong fishy smell, both of which can negatively impact the appearance and acceptability of the final product. Notably, in this study, the addition of seaweed to dark chocolate did not result in any perceptible change in its appearance (Debbarma et al. 2024). This outcome highlights the effectiveness of the washing and drying processes applied to the raw seaweed, which successfully reduced pigment compounds. Moreover, the dark chocolate itself helped mask the color of the seaweed, further maintaining the product's visual appeal. These results were in agreement with those reported by Mindarwati et al. (2024), who incorporated 30%–40% rehydrated *Gracilaria* sp. from West Java, Indonesia, into a snack product. In their study, the seaweed was soaked in a 0.5% citric acid solution at a ratio of 1:3 (seaweed (w) to citric acid solution (v)) for 30 min. This treatment, which combined washing and a mild acid soak, acted as an effective bleaching step that broke down cell walls and significantly reduced the seaweed's fishy smell. It also successfully removed pigment compounds such as carotenoids and phycoerythrin. As a result, the treated seaweed exhibited a neutral or white color, minimizing any visual impact on the final product (Mindarwati et al. 2024). In the texture profile analysis of the dark chocolate samples, parameters such as hardness, springiness, cohesiveness, chewiness, and gumminess were measured using a three‐point bend rig in a texture analyzer to assess the breaking strength and deformation of the chocolate (Debbarma et al. 2024). In the texture profile analysis of dark chocolate samples, parameters such as hardness, springiness, cohesiveness, chewiness, and gumminess were measured using a three‐point bend rig in a texture analyzer to assess the breaking strength and deformation of the chocolate (Debbarma et al. 2024). The incorporation of seaweed increased the breaking strength and firmness, particularly at higher concentrations. Chocolates enriched with *G. edulis* exhibited higher hardness and chewiness compared to those containing *K. alvarezii*. In contrast, lower amounts of *K. alvarezii* (1%–2.5%) had little effect on the textural properties. Parameters such as adhesiveness, deformation, cohesiveness, and springiness remained largely unchanged across all samples. When seaweed was added at low concentrations (around 1%–2.5%), this level may not be sufficient to cause significant changes in nutrient content or texture. The increased hardness in chocolates with *G. edulis* is likely due to its higher protein and fiber content compared to *K. alvarezii*, which may help reinforce the product's structure (Debbarma et al. 2024). Additionally, the increased hardness of the product after adding seaweed may be attributed to its role as a binding agent in food formulations. Seaweed's polysaccharides are crucial in maintaining the cohesion of ingredients, which can help decrease brittleness and contribute to a firmer texture. Moreover, interactions between seaweed and other components, such as starches or proteins, may also influence the hardness and brittleness of the final product (Mindarwati et al. 2024).

One study aimed at masking the taste of seaweed in jam used *Gracilaria changii* to address its strong oceanic flavor and aftertaste (Syahira et al. 2024). The researchers tested various combinations of traditional Southeast Asian palm sugars, including gula apong (smoky, caramel‐like), gula kabong (earthy), and gula melaka (sweet), as natural sweeteners that can replace refined sugar. They found that the 50:50 blend of gula apong and gula kabong was most effective in masking the seaweed's taste, likely due to the flavor‐enhancing compounds formed during heating, such as those from Maillard reactions. Additionally, soaking the dried seaweed overnight in hot water with lime and salt helped reduce its oceanic notes. This approach successfully improved the jam's taste, aroma, and overall acceptability (Syahira et al. 2024). Similarly, other researchers attempted to mask the taste of *Gracilaria* sp. seaweed in jelly candy by combining it with mango juice. The ingredients used for making seaweed jelly candy included 250 mL of water, 240 g of sugar, 10 g of agar powder, and 10 g of *Gracilaria* sp. red algae seaweed powder (Luthfiah et al. 2024). Mango juice was added at different treatment levels: 8.93%, 12.5%, 16.07%, and a control without mango juice. The sensory evaluation of seaweed jelly candy indicated that the 16.07% mango juice treatment received the highest ratings for odor and flavor, whereas the version without mango juice had the lowest flavor score but the best texture score. Color was most preferred in the 12.5% and 16.07% mango juice treatments, while the 8.93% treatment had the lowest rating. Mango juice significantly influenced odor, flavor, and color, but texture differences among samples were minimal. However, the study did not investigate whether the acidity of mango juice affected the gelling behavior.

A study by Charoenphun et al. (2025) examined the effects of different concentrations of *Gracilaria fisheri* powder, a red macroalga in the phylum Rhodophyta, used as a gelling agent at 0.2%, 0.4%, 0.6%, 0.8%, and 1.0% in strawberry‐based drinking jelly. Their results revealed that red seaweed significantly influenced the jelly's color, texture, and flavor. Higher concentrations of the red seaweed increased the L* and a* values, enhancing brightness and redness, while the b* values declined. Lower concentrations of the seaweed showed minimal variation in lightness, whereas moderate and high levels significantly improved color intensity. The 0.8% concentration provided the best balance of color attributes, enhancing the jelly's aesthetic appeal. Texture analysis showed that increasing seaweed concentration strengthened the gel network, improving the structure and homogeneity of the drinking jelly. However, low concentrations resulted in weak, liquid‐like gels, while moderate levels (0.8%) formed a well‐defined, continuous gel matrix with optimal texture. At 1.0%, over‐gelation reduced drinkability (Charoenphun et al. 2025). While color and texture were optimized at 0.8%, sensory evaluations were not conducted. Although volatile compounds such as 1‐octanol (citrus‐like) and methyl anthranilate (grape‐like) were detected in the final product, the seaweed flavour remained undetectable, likely due to the low concentration of seaweed. The gelling properties of this seaweed were attributed to sulfated galactans, also known as agarans, which had stabilizing and thickening capabilities (Zhang et al. 2019).

A study assessed the production and sensory evaluation of butterfly pea flower (0.17 g) and *Eucheuma cottonii* (*Kappaphycus alverezii*) (7 g) seaweed‐based jelly (Sukmawati et al. 2024). The impact of seaweed drying temperatures (50°C, 60°C, 70°C) on color, taste, and texture was analyzed using a 5‐point hedonic scale for sensory evaluations and Hunter's colorimeter for color measurements. Drying temperature affected the sensory properties, with color differences observed, though not statistically significant. Higher drying temperatures led to the breakdown of chlorophyll in the seaweed structure, which could alter the color and result in a noticeable decrease in brightness. Additionally, the incorporation of butterfly pea flowers, rich in anthocyanins, into the seaweed jelly base triggered further transformation, causing a distinct shift toward a purplish tint. Aroma remained unchanged across temperatures, while taste was significantly influenced, with 60°C receiving the highest preference. Statistical analysis revealed significant textural differences between samples dried at 50°C, 60°C, and 70°C. Drying at 50°C yielded the most preferred gelatinous texture, with panelists giving it an average score of 3.80. In contrast, higher drying temperatures produced a gelatinous consistency with reduced density and chewiness. These variations are likely related to carrageenan, a seaweed polysaccharide known for its thickening and gelling properties, which plays a key role in texture development (Bukhari et al. 2023). However, the study by Sukmawati et al. did not evaluate the carrageenan content or gel strength of the seaweed. Without data on sulfation patterns or molecular weight distribution, it is insufficient to attribute the observed texture differences solely to drying temperature.

The sensory and texture evaluation of *Eucheuma cottonii* (*Kappaphycus alvarezii*) seaweed jelly candies (seaweed: water = 1:5) was conducted with four different treatments of red ginger extract (0%, 40%, 50%, and 60%) by twenty panelists (Amalia et al. 2021). The results showed that all treatments were well‐received, with no significant differences in appearance across the treatments. The addition of red ginger extract enhanced both the aroma and flavor, with higher concentrations masking the seaweed smell and imparting a distinct ginger taste. The highest taste rating was given to the 50% red ginger extract treatment, which combined sweetness with a strong ginger flavor. The control treatment (0%) received the lowest aroma and taste scores, offering only a sweet flavor with a mild seaweed scent. The texture analysis of the jelly candy across all treatments revealed a chewy, dense, and slightly sandy consistency. The chewiness was attributed to the presence of *E. cottonii* seaweed, which contains hydrocolloid compounds, primarily carrageenan. With a carrageenan content of 65.75%, *E. cottonii* produces kappa carrageenan, known for forming strong and dense gels. The sandy texture resulted from sugar crystallization on the candy's surface during the drying process. Additionally, the low water content of the candies contributed to their chewiness, as reduced moisture prevented excessive softening of the gel structure. Thus, high carrageenan content and controlled water levels are key factors in achieving the desirable texture of jelly candy. However, flavor‐masking strategies using ginger extract in jelly candy should be combined with crystallization control; otherwise, the improved aroma and flavor may be compromised by undesirable texture. Researchers also analyzed the sensory evaluation of milk chocolate bars with varying amounts of *E. cottonii* seaweed flour: control (no seaweed flour), 5%, 10%, and 15% additions (Stefani et al. 2019). The panelists rated taste as the most important factor, followed by texture, appearance, and aroma. Based on these preferences, the milk chocolate bar with 5% seaweed flour received the highest score, making it the most favored by the panelists. The control (without seaweed flour) ranked second, while the treatments with 10% and 15% seaweed flour were less preferred. Similarly, chocolate pudding was made by combining *E. cottonii* seaweed and tofu dregs in varying ratios of 5:25, 10:20, 15:15, 20:10, and 25:5, along with 100 mL of water, 17 g of skim milk, 8 g of cocoa powder, and 30 g of sugar in each batch (Sukotjo et al. 2020). The sensory evaluation revealed that the chocolate pudding with a 15:15 seaweed to tofu dregs ratio was the most preferred overall, due to its favorable texture, color, and aroma. Its taste was comparable to the 5:25 and 10:20 ratios, making the 15:15 formulation the best choice. The sensory evaluation revealed that the chocolate pudding with a 15:15 seaweed‐to‐tofu dregs ratio was the most preferred overall due to its favorable texture, color, and aroma. Its taste was similar to the 5:25 and 10:20 ratios, making the 15:15 formulation the best option. In both studies, the ideal seaweed inclusion levels appeared to be around 5% for chocolate bars and 15% for pudding. Beyond these levels, the strong seaweed flavor or changes in texture began to outweigh the benefits. However, as instrumental texture analysis (measuring hardness, viscosity, and elasticity) was not conducted, the texture assessments relied entirely on panelists’ subjective opinions. The chocolate pudding's dense yet soft texture could be attributed to carrageenan in the seaweed, whose gel‐like consistency results from the double‐helix structure of its polymer. The addition of seaweed flour also enhanced chewiness and intensified the aroma and flavor (Widati et al. 2021).

Researchers obtained an unidentified brown seaweed from Nirwana Beach, West Sumatra, using 25 g of dried seaweed in 375 mL of water (Faridah 2019). The extract was then incorporated into jelly candies at concentrations of 0%, 5%, 10%, and 15%. Sensory evaluation was conducted by 30 panelists, who rated attributes such as shape and seaweed flavor on a 0–4 scale. The sensory evaluation revealed that jelly candies with brown seaweed extracts were well‐accepted, as their characteristics were influenced by the presence of fucoxanthin and alginate in the brown seaweed. As the concentration of the seaweed extract increased, the color and flavor ratings improved, with the 15% extract providing the most intense color and flavor. The addition of the seaweed did not impact the shape or uniformity of the taste. The jelly candy with 15% seaweed extract had the highest overall acceptability, boosting the product's appeal. However, the study did not discuss potential interactions between seaweed‐derived alginate and the agar used in the formulation (9 g/100 g), which could influence gel strength and syneresis.

A study evaluated the sensory characteristics of three seaweed jelly candies prepared with varying ratios of *Caulerpa* sp. (seaweed) to sugar (w/w): 1:0.5, 1:1, and 1:1.5 (Tapotubun et al. 2018). The candies were assessed for color, flavor, texture, and smell. The 1:1.5 ratio stood out as the most preferred due to its well‐balanced taste, pleasant texture (elastic, solid, and slightly rough), and a pleasing scent that combined the seaweed's natural ocean‐like aroma with the sweetness of sugar, making it the optimal choice for jelly candy production. The jelly had a sweet and slightly salty flavor, with the higher sugar content decreasing the saltiness. Meanwhile, the 1:1 ratio of seaweed to sugar was favored for its green color, which was attributed to the chlorophyll in the seaweed. However, it is important to note that high sugar content poses health concerns, particularly in products aimed at offering functional or health‐promoting benefits. If the objective is to create a functional jelly candy, replacing refined sugar with sugar alcohols like sorbitol or honey could be advantageous. Such alternatives provide sweetness with lower caloric value and reduced glycemic impact, supporting both the functional and nutritional goals of the product. For example, researchers used the extract of fresh *Caulerpa* sp. to produce candies with sorbitol and a very low sugar concentration (Fransiska et al. 2020). The extract was combined with distilled water in different ratios (0:4, 1:3, 2:2, 3:1, and 4:0), along with 5% gelatin, 3% sorbitol, 1% sugar, and mint and guava flavors to mask the seaweed odor. The mixture was heated, thickened at 90°C, cooled to 70°C, then poured into 8 cm Teflon molds and stored for two days at 15°C before being packaged as candy. Sensory evaluation showed that a 2:2 ratio of seaweed extract to water achieved the best balance of texture, flavor, aroma, and overall acceptability. This suggests that a moderate amount of extract enhances consumer acceptance without overwhelming the candy's sensory profile. Additionally, the levels of reducing sugars in the final product remained within acceptable limits (up to 25% for jelly candy).

Incorporating seaweed or seaweed‐derived ingredients into confectionery products offers a range of sensory benefits and challenges. Natural pigments from seaweeds, such as fucoxanthin and chlorophyll, provide safe alternatives to artificial dyes, although their lower stability and intensity compared to synthetic dyes must be considered. Seaweed's impact on the color, texture, and flavor of products like gummies, chocolates, and jellies varies depending on the type and concentration of seaweed used. While sensory evaluation suggests that small additions of seaweed do not significantly affect consumer acceptance, higher concentrations can influence texture and flavor, with some varieties imparting a fishy smell or modifying texture attributes such as hardness and chewiness. Additionally, seaweed's natural polysaccharides, such as carrageenan, play a key role in maintaining the texture and stability of these products. Accurately measuring the polysaccharide content (such as carrageenan, agarose, and alginate) of seaweed, along with their functional properties (such as gel strength and viscosity), is essential. Without this data, formulating plant‐based confectionery products may lead to inconsistent or unpredictable textures. Overall, developing plant‐based, seaweed‐fortified confectionery should follow a holistic approach: (1) define the target nutritional claim, such as high in fiber or low in fat; (2) select the appropriate seaweed species and form, such as powder, extract, or seaweed‐derived ingredients, based on its ingredient profile; (3) optimize the inclusion level using a design of experiments approach, considering color, taste, flavor, and texture; (4) conduct texture analysis and in vivo assays, such as rheology and bioavailability testing through human trials, to assess the functional performance and nutrient absorption; and (5) validate consumer acceptance through rigorous sensory evaluation panels.

## 5 Challenges and Limitations

A key challenge is developing a sensory profile that aligns with consumer expectations while incorporating seaweed into confectionery. As seaweed is not a traditional ingredient in most Western markets, its unfamiliar flavor and potential skepticism may limit acceptance. This makes sensory evaluation essential in product development, especially when introducing novel ingredients to these markets (Salgado et al. 2023). Sensory quality is crucial to a food product's success, but factors such as familiarity, novelty, and branding also shape consumer perception (Tuorila 2007). Effective product development strategies must balance masking off‐flavors with appealing to consumer preferences. While health benefits can enhance a product's value, they hold little influence if the sensory experience is unappealing. Therefore, sensory analysis and consumer response data are essential for predicting product success (Tuorila and Cardello 2002). For instance, dark chocolate effectively masked the flavor and odor of seaweed in chocolates containing up to 7.5% (w/w) *Gracilaria edulis* and *Kappaphycus alvarezii* dried red seaweed powder, suggesting potential for consumer acceptance (Debbarma et al. 2024). A sensory analysis study in Portugal showed that milk chocolate with 4% kombu (MK) and ruby chocolate with 3% nori (RN) were favored for their familiar flavors and pleasant texture, leading to positive emotions such as satisfaction (Salgado et al. 2024). White chocolate with 3% sea lettuce (WS) had a unique flavor profile, evoking mixed emotional responses, including disappointment and lack of calm, though it was also described as adventurous by some. Overall, MK and RN were better liked, while WS had more varied emotional associations. Seaweeds have the potential to be incorporated into chocolate, enhancing both its nutritional value and organoleptic diversity. This could serve as an effective strategy to introduce seaweed into new markets, offering consumers a unique and health‐conscious alternative.

Technological advancements represent a key challenge in the sustainable use of natural resources. While many countries, particularly in Europe, are working to reduce dependence on unsustainable materials, introduce healthier natural products, and support coastal communities, several obstacles remain. One of the main hurdles is the development of innovative and cost‐efficient processing technologies to maximize the potential of underutilized feedstocks like seaweed. Incorporating these value‐added ingredients into functional foods, such as confectionery products, presents both a challenge and an opportunity (de Lima Xavier et al. 2024).

In addition to technological challenges, variability in the biochemical constituents of seaweed, such as polyphenols, carbohydrates, and proteins, adds another layer of complexity. These constituents fluctuate based on harvest time, environmental conditions, and location, leading to inconsistencies (Suresh Kumar et al. 2021). While cultivated seaweed offers better control over quality and supply, wild‐harvested seaweed may face sustainability and consistency issues. Furthermore, low consumer awareness of seaweed's health benefits, along with concerns about contaminants, food safety, and anti‐nutritional factors, remains a barrier to its use in food products (Garcia‐Vaquero et al. 2017; Samarathunga et al. 2023).

The shortage of seaweed presents another challenge, as seaweed aquaculture makes up 97% of global production, while wild harvesting contributes only a minor share. However, aquaculture is concentrated in specific regions and limited to five main genera, with the following production contributions: *Laminaria*/*Saccharina* at 35.4%, *Kappaphycus/Eucheuma* at 33.5%, *Gracilaria* at 10.5%, *Porphyra*/*Pyropia* at 8.6%, and *Undaria* at 7.4%. Asia leads in seaweed aquaculture, accounting for over 97% of global production (WHO 2022). The dependence on a limited number of genera and localized production areas presents considerable challenges to the global seaweed supply chain.

Another significant challenge in incorporating seaweed into food products, such as confectionery, is the presence of toxic metabolites and contaminants. Numerous toxic compounds have been identified in seaweeds that pose potential health risks. One such compound is domoic acid, a potent neurotoxin responsible for amnesic shellfish poisoning (Zabaglo et al. 2016). Domoic acid was first discovered in the red seaweed *Chondria armata* and has since been found in other red seaweed species. Although small doses of domoic acid (0.04–0.8 mg/kg body weight) caused no observable effects in a study involving Japanese children, the compound is still considered hazardous to human health (Banach et al. 2020). Another neurotoxin, kainic acid, occurs naturally in red seaweeds such as *Palmaria palmata* (commonly known as dulse), with concentrations ranging from undetectable to over 10,000 mg/kg d.w. Despite its toxicity at elevated levels, no formal regulatory limits have yet been established for kainic acid in food‐grade seaweeds (Banach et al. 2020). Several species from the red seaweed genus *Gracilaria* have been implicated in poisoning incidents and fatalities, particularly in the Pacific region (Cheney 2016). *Gracilaria verrucosa* and *G. chorda*, for example, have been linked to symptoms such as gastrointestinal distress, internal bleeding, and, in severe cases, fatal hypotensive shock effects likely attributed to prostaglandins (M. S. Kumar and Sharma 2021). Other species, such as *Gracilaria coronopifolia* and *edulis* (also known as *Polycavernosa tsudai*), have caused poisoning characterized by muscle spasms, neurological impairment, and numbness. Identified toxins in these cases include aplysiatoxin, debromoaplysiatoxin, manauealides, malyngamides, and polycavernosides. Similarly, the red seaweed *Acanthophora specifera* has been associated with fatal outbreaks, displaying symptoms nearly identical to those of *G. edulis*, suggesting the presence of related toxic compounds. In the case of green seaweeds, particularly those in the genus *Caulerpa*, several bioactive substances have been noted for their toxicological effects (Cheney 2016). For instance, caulerpicin from *Caulerpa racemosa* causes oral numbness and cold sensations in the limbs. Caulerpin has sedative effects and may lead to respiratory difficulties and loss of coordination. Another compound, caulerpenyne, is a cytotoxic and neurotoxic metabolite, raising concerns about its potential impact on human health. Seaweeds can also harbor harmful microorganisms, such as cholera (Løvdal et al. 2021) and dinoflagellates (Imai 2015). Proper cleaning of seaweed is crucial to prevent contamination (A. Kumar et al. 2023).

Allergenic potential is another important concern. Clinical evidence has revealed immunoglobulin E (IgE)‐mediated allergic reactions to several seaweed species. Red algae such as *Porphyra*, *Chondrus crispus*, and *Palmaria palmata*, as well as green algae like *Ulva*, have been identified as allergenic in sensitized individuals (Daniel and Tolentino 2023; Garciarena et al. 2022; James et al. 2023). Moreover, dried seaweed products like nori have been found to contain proteins structurally, like known crustacean allergens (M. S. Kumar and Sharma 2021). This cross‐reactivity suggests that individuals with shellfish allergies could experience severe allergic responses after consuming certain seaweed‐derived products. Additionally, seaweed is prone to microplastic contamination, which may carry harmful chemicals like phthalates and bisphenol A (Kibria et al. 2022; Padervand et al. 2020). Even after washing, microplastics can persist in processed products like nori, underlining the need for more effective cleaning methods (Li et al. 2020). Furthermore, seaweeds absorb heavy metals such as arsenic, mercury, and cadmium from their environment, although often within safe limits (Lindenmayer et al. 2023). Seaweed is rich in iodine, which is crucial for health, but consuming too much, particularly from species like *Saccharina latissima* (Nielsen et al. 2020), *Fucus vesiculosus*, and *Laminaria digitata* (Nitschke et al. 2018), can result in toxicity. Implementing standardized cleaning, refining processing methods, and carefully selecting species and cultivation practices are crucial. Effective processing and ingredient selection can help minimize contaminants, ensuring the safety and quality of seaweed‐based confectionery products. While seaweed offers multiple functional benefits, overcoming challenges related to sensory attributes, process standardization, and regulatory compliance is critical for its successful commercialization in the confectionery sector.

## 6 Regulatory Frameworks for Seaweed in Confectionery Products

Despite the growing use and nutritional appeal of seaweed in food products such as confectionery, there remains a notable lack of comprehensive global regulation addressing its food safety (FAO; WHO; 2022). Currently, no Codex Alimentarius standard or dedicated code of practice specifically governs seaweed. The Food and Agriculture Organization (FAO) has highlighted this regulatory gap, noting that the absence of a unified international framework creates barriers to global trade and consistent safety assurance (Baghel et al. 2023).

To manage these concerns, several countries have developed their own regulations. In the United States, the Food and Drug Administration (FDA) classifies unprocessed seaweed as a raw agricultural commodity (RAC), subjecting it to the Federal Food, Drug, and Cosmetic Act (Baghel et al. 2023). This classification influences how seaweed is processed and incorporated into products such as confectionery. Within the European Union (EU), seaweed is regulated under a complex and evolving framework (FAO; WHO; 2022). Food safety is primarily governed by Regulation (EC) No 852/2004 on food hygiene, which requires the implementation of Hazard Analysis and Critical Control Point (HACCP) systems in seaweed‐related food production, a method endorsed by both the World Health Organization and the Codex Alimentarius Commission (Hofmann et al. 2024; Løvdal et al. 2021).

The EU recently updated its Novel Food Status Catalogue, recognizing more than 20 seaweed species, including *Gelidium amansii*, *Erythroglossum laciniatum*, *Laminaria hyperborea*, *Pyropia yezoensis*, *Porphyra dioica*, *Saccharina japonica*, and *Ulva intestinalis* as non‐novel foods (European Commission 2024). This permits their sale without pre‐market authorization, reducing regulatory costs by approximately €10 million and easing market access, especially for exporters from low‐ and middle‐income countries. Other species, such as *Eucheuma denticulatum*, *Alsidium helminthochorton*, *Gracilaria gracilis*, *Ecklonia cava*, and *Macrocystis pyrifera* are classified as non‐novel only when used in food supplements, with possible restrictions for other uses. These changes support the EU's 2022 algae strategy, which promotes sector growth and innovation (AGRINFO 2025). Novel food containing phlorotannins, extracted from the edible brown seaweed *Ecklonia cava*, is authorized with specified maximum daily intake limits of 163 mg for adolescents aged 12 to 14, 230 mg for those over 14, and 263 mg for adults. Products must be labeled as “Ecklonia cava Phlorotannins.” Supplements containing it should include warnings advising against use by children under 12, individuals with or at risk of thyroid disease, and those taking other iodine supplements (European Commission 2018). Similarly, novel foods containing fucoidan extracts derived from *Fucus vesiculosus* and *Undaria pinnatifida* are permitted for use in foods and food supplements, following extraction through aqueous acid solutions and filtration without organic solvents. They must be labelled respectively as “Fucoidan extract from seaweed *F. vesiculosus*” or “Fucoidan extract from seaweed *U. pinnatifida*,” with a maximum daily intake of 250 mg (European Union 2017).

Contaminant control is a key regulatory focus due to seaweed's ability to absorb heavy metals and environmental pollutants. The European Food Safety Authority (EFSA) has identified seaweed as a potential emerging risk (Sá Monteiro et al. 2019). Although Commission Regulation (EC) No 1881/2006 sets maximum levels for arsenic, cadmium, and lead in various foods (European Union 2018), specific maximum levels for seaweed remain limited, except in food supplements primarily derived from it. For example, EU Regulation 2021/1323 establishes a cadmium limit of 3 mg/kg (wet weight) for seaweed‐based supplements (European Union 2021). Commission Regulation (EU) No 1275/2013 limits arsenic in seaweed meal and feed materials to 40 mg/kg at 12% moisture content (EU 2013). France applies stricter national limits for dried seaweed, including lead (5 mg/kg), cadmium (0.5 mg/kg), tin (5 mg/kg), mercury (0.1 mg/kg), inorganic arsenic (5 mg/kg), and iodine (2000 mg/kg) (Purcell‐Meyerink et al. 2021).

Iodine regulations vary widely. Germany, France, and Nordic countries set maximum levels at 20, 2000, and 115 mg/kg (d.w.), respectively (Hofmann et al. 2024), while the EU and China lack harmonized standards. This inconsistency has led to multiple alerts in the European Rapid Alert System for Food and Feed (RASFF). A search on June 10, 2025, found 43 notifications concerning excessive iodine levels linked to seaweed and algae.

All seaweed‐based products marketed in the EU must comply with Regulation (EU) 1169/2011 on food labeling (Garcia‐Vaquero and Hayes 2016). However, concerns persist about insufficient labeling of minerals, heavy metals, and safe consumption guidelines (Leandro et al. 2020). Seaweed‐enriched confectionery can carry health claims like “mineral‐rich,” “fat‐free,” “low sugar,” “gluten‐free,” and “low calorie,” provided they meet EFSA standards (Matos et al. 2021). Despite EFSA's ongoing efforts, establishing comprehensive, consistent labeling remains a challenge.

Outside the EU, regulations differ. Australia limits iodine and arsenic in imported seaweed; Chile regulates algae extracts for human and animal consumption; Japan monitors heavy metals and issues safety advisories; Norway and Korea require pre‐market authorization and risk assessments for novel seaweed products. Similarly, the U.S. FDA evaluates seaweed ingredients to ensure compliance with safety standards (Codex Alimentarius Commission 2022). Despite regulatory advances, the seaweed industry faces challenges including lack of standardized methods for bioactive compound analysis, inconsistent product quality, and concerns about heavy metals and natural toxins. Navigating diverse systems such as the EU's Novel Food classification and the U.S. FDA's GRAS designation complicates product approval and commercialization. Therefore, harmonized international regulations and clearer safety guidelines are urgently needed to support seaweed sector growth. Addressing these gaps will facilitate global market access, ensure consumer safety, and promote sustainable innovation in seaweed‐based foods, including functional confectionery.

## 7 Sustainability and Economic Aspects

The global seaweed market, valued at $7.0 billion in 2023, is expected to grow to $16.1 billion by 2033 (Makode and Deshmukh 2024). This growth is driven by increasing interest in using seaweed as a food source, with the market in Europe, North America, and Australasia experiencing an annual growth rate of 7%–10% (Food Safety Authority of Ireland 2020). The current market for seaweed‐based confectionery is relatively niche, primarily driven by health‐conscious consumers and those seeking novel food experiences. Establishing a competitive price point that reflects the added nutritional and functional benefits of seaweed while remaining attractive to consumers is essential for market success (Pujiastuti et al. 2021). The creation of value‐added products is determined not only by the number of processing stages, additional inputs, and technology employed, but also by the market demand—specifically, how much the final consumer is willing to pay. This is reflected in consumer spending, with processed products being purchased at rates six times higher than fresh products (Sudarwati et al. 2020). However, a recent study at the University of Limerick involving a sample of 50 participants aged 18 to 45 in Ireland examined the purchase intention of gummies containing Atlantic wakame and sea lettuce extracts (23 g/100 g of gummies) (Xavier et al. 2024). The study found no significant difference between the control (without seaweed extract) gummies and sea lettuce gummies, while Atlantic wakame gummies were comparable to sea lettuce gummies. This suggested that low consumer awareness of seaweed's benefits might have limited purchase intention, highlighting the need for better education and marketing. In addition, research suggested that refining the production process and using activity‐based costing (ABC), a method that assigns costs to products based on the activities involved in production, could enhance marketing efforts (Zaid et al. 2019). In the seaweed jelly industry, produced in rural villages, products were transported to wholesalers for further refining, with prices increasing at each stage. However, profit margins remained limited due to high raw material costs. By implementing ABC, businesses could more accurately allocate costs, improve decision‐making, and enhance pricing strategies, ultimately boosting the appeal of seaweed‐based gummies.

## 8 Future Prospects and Research Directions

Future research should prioritize the development of cost‐efficient and scalable processing methods, including optimized extraction and encapsulation techniques, to preserve seaweed's bioactive compounds, such as phlorotannins, while maintaining appealing sensory attributes. Exploring underutilized seaweed species to identify bioactive compounds, such as phytosterols, ulvans, laminarans, fucoidans, and natural pigments like fucoxanthin, is also recommended. Additionally, the effects of adding these ingredients on the texture, color, and sensory properties of the final product could be further investigated. Pre‐treatment and bleaching conditions, along with the addition of edible acids such as citric acid to remove color or mask flavor, should also be explored. The final product could then be tested during storage to evaluate microbial load, texture, and pigment stability. Standardizing cultivation and processing protocols will be crucial to ensuring product consistency and safety. Addressing regulatory challenges, increasing consumer awareness, evaluating the sustainability of scaled production, and conducting long‐term human trials to validate health benefits and determine effective dosages will be essential to establishing seaweed‐based confectionery as a competitive and sustainable alternative in the market.

## 9 Conclusion

Seaweed offers a promising plant‐based alternative to animal‐derived gelatin, enhancing the functionality and nutritional profile of confectionery products while supporting sustainability. Seaweed's hydrocolloids, such as agar, carrageenan, and alginate, provide gelling, thickening, stabilizing, and moisture‐retention properties, while its rich content of fiber, antioxidants, minerals, and unique bioactive compounds further boosts the nutritional value of products such as gummies, jelly drinks, candies, and chocolates. Research highlights improvements in protein and fiber content, along with biological activities such as antioxidant, anti‐obesity, anti‐anemia activity, and photoprotective effects. As a renewable resource with a low ecological impact, seaweed contributes to global sustainability goals and meets the growing consumer demand for eco‐friendly, clean‐label, and plant‐based foods. However, to achieve successful market integration, it is essential to address challenges related to sensory acceptance, processing technology, and regulatory requirements. Continued efforts in processing innovation, validation of health claims through in vivo scientific studies, and increased consumer awareness will be key to advancing this field. Seaweed‐enriched confectionery products hold strong potential to transform the category from traditional confectionery to innovative plant‐based and sustainable alternatives, positioning them as future foods.

## Author Contributions

**Nima Mohammadi**: conceptualization, investigation, writing – original draft, methodology, visualization, writing – review and editing, project administration, supervision, resources, software, data curation, validation. **Nikoo Ostovar**: methodology, investigation, writing – original draft, formal analysis, data curation, writing – review and editing.

## Conflicts of Interest

The authors declare no conflicts of interest.
